# FAMetA: a mass isotopologue-based tool for the comprehensive analysis of fatty acid metabolism

**DOI:** 10.1093/bib/bbad064

**Published:** 2023-03-01

**Authors:** María I Alcoriza-Balaguer, Juan C García-Cañaveras, Marta Benet, Oscar Juan-Vidal, Agustín Lahoz

**Affiliations:** Biomarkers and Precision Medicine Unit, Medical Research Institute-Hospital La Fe, Av. Fernando Abril Martorell 106, Valencia 46026, Spain; Biomarkers and Precision Medicine Unit, Medical Research Institute-Hospital La Fe, Av. Fernando Abril Martorell 106, Valencia 46026, Spain; Biomarkers and Precision Medicine Unit, Medical Research Institute-Hospital La Fe, Av. Fernando Abril Martorell 106, Valencia 46026, Spain; Biomarkers and Precision Medicine Unit, Medical Research Institute-Hospital La Fe, Av. Fernando Abril Martorell 106, Valencia 46026, Spain; Biomarkers and Precision Medicine Unit, Medical Research Institute-Hospital La Fe, Av. Fernando Abril Martorell 106, Valencia 46026, Spain; Analytical Unit, Medical Research Institute-Hospital La Fe, Av. Fernando Abril Martorell 106, Valencia 46026, Spain

**Keywords:** fatty acids, stable isotopes, lipid metabolism, mass spectrometry, inhibitors

## Abstract

The use of stable isotope tracers and mass spectrometry (MS) is the gold standard method for the analysis of fatty acid (FA) metabolism. Yet, current state-of-the-art tools provide limited and difficult-to-interpret information about FA biosynthetic routes. Here we present FAMetA, an R package and a web-based application (www.fameta.es) that uses ^13^C mass isotopologue profiles to estimate FA import, *de novo* lipogenesis, elongation and desaturation in a user-friendly platform. The FAMetA workflow covers the required functionalities needed for MS data analyses. To illustrate its utility, different *in vitro* and *in vivo* experimental settings are used in which FA metabolism is modified. Thanks to the comprehensive characterization of FA biosynthesis and the easy-to-interpret graphical representations compared to previous tools, FAMetA discloses unnoticed insights into how cells reprogram their FA metabolism and, when combined with FASN, SCD1 and FADS2 inhibitors, it enables the identification of new FAs by the metabolic reconstruction of their synthesis route.

## Introduction

Fatty acids (FAs) are key metabolites that play a central role in cellular biology. FAs act as building blocks for the synthesis of complex lipids or as a source of energy, but also as signaling molecules [1]. Dysregulated FA metabolism has been associated with many of the most prevalent diseases, including obesity [2], type 2 diabetes [3], non-alcoholic fatty liver disease [4], or cancer [5]. FAs can be either synthesized *de novo* inside cells or imported from external sources. The main product of *de novo* lipogenesis (DNL) is palmitic acid [FA(16:0)], which results from the condensation of acetyl-CoA molecules through the enzymatic action of acetyl-CoA carboxylase (ACACA/B) and FA synthase (FASN). The acetyl-CoA pool is generated via ATP citrate lyase (ACLY) from citrate that can, in turn, be produced from several carbon sources (i.e. glucose, glutamine, amino acids, FAs), or from acetate via acetyl-CoA synthetases (ACSS1/2) [6]. Linoleic [FA(18:2n6)] and γ-linolenic acid [FA(18:3n3)] are essential FAs that must be exogenously acquired. Free FA import occurs by either passive diffusion or the action of translocases like CD36 and FA transport proteins (FATPs). FAs can be elongated via very long-chain FA proteins (ELOVL1-7). They can also be desaturated via the action of stearoyl-CoA desaturases 1/5 (SCD1/5) and FA desaturases 1/2 (FADS1/2) enzymes [1, 5]. The wide variety of FAs required for the cellular functioning results from these transformations.

Stable-isotope tracing combined with mass spectrometry (MS)-based detection is a widespread method for interrogating FA metabolism. The total FA synthesis rate can be estimated by using D_2_O, which labels FAs through direct solvent incorporation and NADPH-mediated hydrogen transfer [7, 8]. Additionally, employing ^13^C-labeled tracer nutrients (e.g. U-^13^C-glucose, U-^13^C-glutamine, U-^13^C-acetate, etc.) allows the total FA synthesis rate and the relative contribution of a given nutrient to be estimated [9]. The framework for FA synthesis data analysis using ^13^C-labeled tracers and MS was initially set up by Mass Isotopomer Distribution Analysis (MIDA) [10] and Isotopomer Spectral Analysis (ISA) [11], which model FA synthesis following the incorporation of n 2-carbon units using multinomial distribution fitting. Unfortunately, these mass isotopologue modeling methods only provide information about the DNL of FAs for which the contribution of elongation is minimal (i.e. FAs of 14 or 16 carbons) [10–12]. ConvISA incorporated one elongation to model up to 18-carbon FAs [13]. Recently, Fatty Acid Source Analysis (FASA) included many elongation steps, which extend the FA species that can be properly modeled up to 26 carbons [14]. However, FASA present some limitations as it assumes *de novo* synthesis up to 26-carbon FAs, and it calculates multiple import-elongation terms (i.e. *IE_n_*, which refers to imported and elongated n times), which does not accurately represent the real biological process. Concerning FA desaturation, only a simple strategy for estimating the desaturation of FA(18:0) to FA(18:1n9) has been described [15]. However, this approach is based on the total labeling of precursor and product FAs [15], and its application to the complete array of desaturations has not yet been explored. Despite these valuable advances, reliable FA elongation calculations are still not fully addressed, whereas systematic desaturation estimations remain unresolved. Moreover, the above-mentioned algorithms were developed for platforms that require computational skills and commercial software; thus, they are not readily accessible to the broad metabolism community. To bridge this gap, we developed FAMetA (Fatty Acid Metabolism Analysis), a mass isotopologue-based tool implemented as an R package and a web-based application that aims to analyze all the biosynthetic reactions within the FA metabolic network. FAMetA provides a complete workflow to analyze MS data and returns easy-to-interpret results that facilitate straightforward FA metabolism analyses and the identification of unknown FAs.

## Results

### FAMetA overview

**Figure 1:**
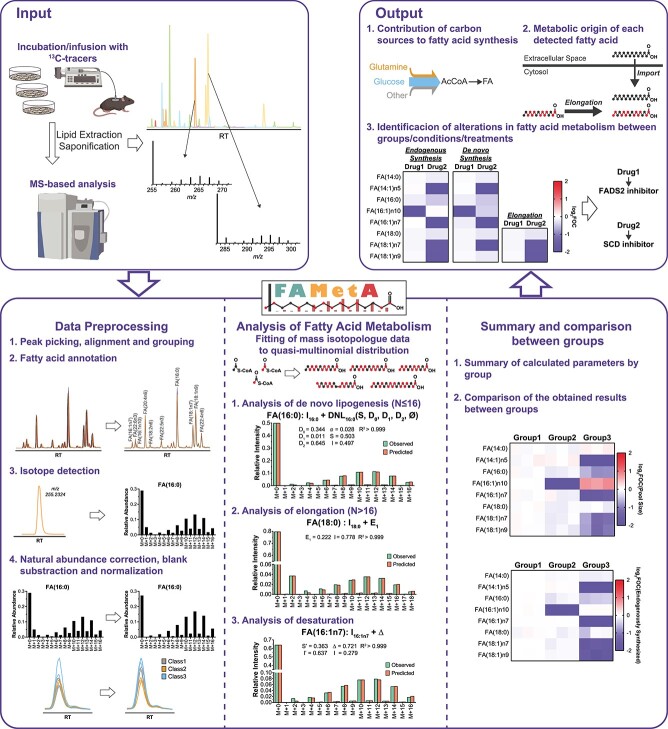
FAMetA workflow. FAMetA is an open-access platform-independent software for estimating FA metabolism based on mass isotopologue data that can be executed locally in an R environment (https://CRAN.R-project.org/package=FAMetA) or online (www.fameta.es). FAMetA uses as input the data generated after incubation/infusion with suitable ^13^C-tracers, based on the LC–MS or GC–MS analysis of FA extracts. Briefly, the FAMetA workflow comprises the following steps: (i) data preprocessing; (ii) analysis of the FA metabolism for each sample and detected FA; and (iii) the combination of these individual results to provide an overview of the FA metabolism network for each condition of interest and to compare them. The analysis of the FA metabolism parameters is based on fitting the experimental mass isotopologue distribution to a quasi-multinomial distribution. For FAs of 14 and 16 carbons, the imported fraction (*I*) and the DNL parameters (i.e. synthesis (S), fractional contribution of the tracer (*D_0_*, *D_1_* and *D_2_*), and overdispersion (Φ)) are calculated. For FAs of more than 16 carbons, the *D_0_*, *D_1_*, *D_2_* and Φ parameters are imported and the sources are described as the import (I) plus the elongation (*E*_n_) of the total pool of the precursor FA [e.g. for FA(18:0), sources are described as *I*_18:0_ + *E*_1_ = *I*_18:0_ + *E*_1_ (*I*_16:0_ + *S*)]. For the FAs that are the result of desaturation, sources are described as the import (*I*), plus desaturation (Δ), where Δ is indirectly estimated according to the synthesis parameters of the precursor (*S* or *E*) and product FAs (*S*′ or *E*’), where Δ = *S*′/*S* or *E*’/*E*. Depending on the experimental design, the most relevant biological outputs to be obtained include the fractional contribution of each tested carbon source, the detailed description of the metabolic origin of each detected FA and the elucidation of an alteration in the FA metabolism between conditions of interest.

**Figure 2:**
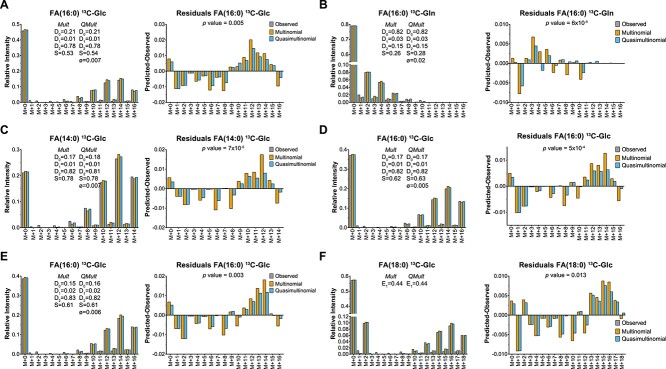
Fitting experimental mass isotopologue FA data to multinomial and quasi-multinomial distributions. (**A**–**B**) FA(16:0) in the A549 cells upon incubation with U-^13^C-glucose (A) or U-^13^C-glutamine (B), data obtained from ref. [18]. (**C**–**D**) FA(14:0) (C) and FA(16:0) (D) in the H1299 cells upon incubation with U-^13^C-glucose, data obtained from ref. [14]. (**E**–**F**) FA(16:0) (E) and FA(18:0) (F) in the MCF7 cells upon incubation with U-^13^C-glucose, data obtained from ref. [13]. For each dataset, the experimental data, the fitting done using the FAMetA algorithm with multinomial or quasi-multinomial distributions, and the residuals are shown. The reported *P*-values correspond to the comparisons between multinomial and quasi-multinomial fitting using a log-likelihood ratio test and right-tailed chi-square distribution.

**Figure 3:**
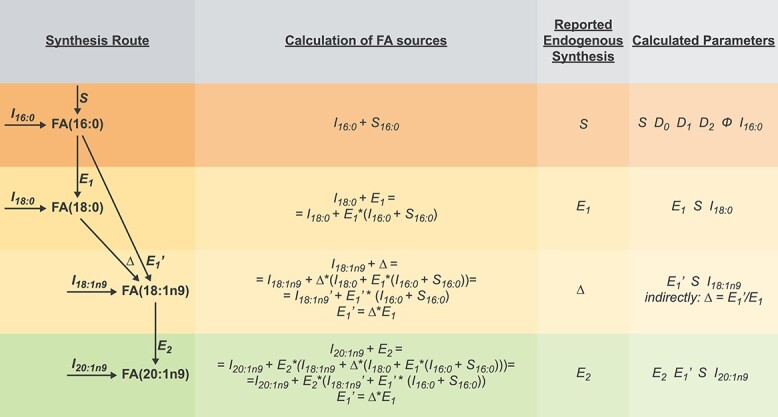
Example of the FAMetA calculations for FA(16:0) to FA(20:1n9). A detailed description of the calculation of FA sources, reported endogenous synthesis and the parameters calculated for the FAs: FA(16:0), FA(18:0), FA(18:1n9), and FA(20:1n9).

FAMetA is an R package (https://CRAN.R-project.org/package=FAMetA) and a web-based platform (https://www.fameta.es) that rely on mass isotopologue distributions from GC–MS or LC–MS to estimate the import (*I*), the synthesis of FA(14:0)/FA(16:0) (*S*), the fractional contribution of the ^13^C-tracer (*D_0_*, *D_1_* and *D_2_*, which represent the acetyl-CoA fraction with 0, 1, or 2 atoms of ^13^C, respectively), the elongation (*E*) and the desaturation (Δ) parameters for the expected network of FA synthesis reactions up to 26 carbons [16] (Figure 1, Supplementary Figure 1). The FAMetA workflow comprises the required functionalities needed, from data preprocessing to group-based comparisons and graphical output (Figure 1, Supplementary Figures 2–3). Mass isotopologue distributions usually show overdispersion, which can be attributed to cellular heterogeneity, time-dependent variations that result from changes in nutrient availability, differences between the various intracellular FA pools (e.g. differences between lipid classes or between FA/lipids located in different organelles), among others. FAMetA implements quasi-multinomial modeling that improves the fitting of mass isotopologue distributions compared with formerly used multinomial modeling [10–14, 17, 18] (Figure 2). Furthermore, this fitting provides the parameter *Φ* that accounts for data overdispersion. For FAs up to 16 carbons, the DNL parameters (*I*, *S*, *Φ* and *D_0_*, *D_1_*, *D_2_*) are estimated. The equations employed to fit the experimental isotopologue distribution are equivalent to those employed by the ISA algorithm [11, 12] if the parameter *Φ* = 0 and if the ISA equations are modified to take into account that the data have been corrected for the natural abundance of ^13^C. Unlike the original ISA algorithm [11, 12], which is designed for calculation of the DNL, or ConvISA [13], which only calculates elongation for FA(18:0), for the FAs of 18–26 carbons, apart from the parameters *S* and *I*, FAMetA estimates up to five elongation terms (*E_n_*, *n* = 1 for 18-carbon to *n* = 5 for 26-carbon FAs). Each elongation term represents the direct estimation of the fraction that comes from the elongation of the total pool of the precursor FA (Figure 3). Compared with previous tools (i.e. FASA, where the synthesis of an FA longer than 16 carbons is described as DNL up to the total length and multiple import-elongation terms are implemented [14]), the way in which elongations are calculated by FAMetA better reflects how FAs are elongated within the cells, which permits the straightforward biological interpretation of the reported elongation parameters. For the FAs that result from the direct desaturation of one precursor FA, Δ is indirectly estimated based on the calculated synthesis parameters of the precursor (*S* or *E*) and the FA of interest (*S′* or *E’*) (i.e. Δ = *S′*/*S* or Δ = *E’*/*E*) (Figure 3). The strategy proposed here is inspired in the simple approach described by Kamphorst et al. [15, 19]. The authors calculate desaturation for FA(18:1n9) based on the total labeling in FA(18:0) and FA(18:1n9). We extend the strategy to the complete set of desaturations within the FA metabolic network and refine the calculation by using an approach that uses the estimated synthesis parameter of interest instead of the total labeling. The use of the complete isotopologue distribution to estimate the substrate and product FA synthesis parameters of interest instead of a single summed value may lead to a more robust and accurate estimation of desaturation. However, the key advance of how FAMetA calculates desaturations compared to the calculation proposed by Kamphorst et al. is the possibility of estimating alterations in concrete desaturation steps from the complete set of parameters calculated for a given FA [e.g. identify alterations in SCD activity between two conditions based on the information obtained for FA(18:1n7), where the double bond is introduced at the 16-carbon level]. Finally, the complete metabolic network of FA synthesis is summarized for each sample and group, and comparisons between groups are made and graphically represented (Figure 1). As in previous tools (i.e. ISA, ConvISA and FASA [11–14]) the *de novo* synthesis parameters (*S*, *E*, Δ) are time-dependent. Therefore, at any given time, such parameters correspond to the fraction of a particular FA that has been *de novo* synthesized up-to-the moment of the sampling, and it corresponds to the actual portion of FA that comes from *de novo* synthesis only if the steady state has been achieved. Accordingly, the import term (*I* = 1 − *S* or 1 − *E_n_*) accounts for both import and pre-existing FAs at any given time and to the actual fraction that is acquired from the exogenous pool when the steady state has been reached. The conditions of metabolic and isotopic steady states are only achieved, or can be closely approximated, if the cells are cultured during a long-enough time to ensure that the pre-existing FA pools can be diluted out while ensuring a nutrient supply that maintains relatively stable concentrations [14, 20]. A key feature of FAMetA compared to previous tools/algorithms is its ease of use and implementation. Although previous tools have been released as Matlab scripts or implemented within complex software that require extra tools for data-preprocessing and graphical representation, FAMetA R package and web-page (www.fameta.es) provide the whole workflow for MS data analysis. A comprehensive comparison of the functions implemented by FAMetA and other available tools is summarized in Supplementary Table 1.

### FAMetA validation

*In silico* mass isotopologue distributions are generated to validate the FAMetA algorithm. To simulate experimental distributions, multiple values covering the expected range for each parameter are used. For each theoretical isotopologue distribution, 10 realizations of Gaussian noise are simulated at four noise levels [0, 2, 5, or 10% relative standard deviation (RSD)]. The generated data are used to calculate the RSD and relative error of each modeled synthesis parameter for the following FAs, which comprise an example of all the reactions included in FAMetA: FA(16:0) (Supplementary Figure 4), FA(18:0) (Supplementary Figure 5), FA(20:0) (Supplementary Figure 6), FA(22:0) (Supplementary Figure 7), FA(24:0) (Supplementary Figure 8), FA(16:1n7) (Supplementary Figure 9) and FA(18:1n9) (Supplementary Figure 9). FAMetA accurately determines the complete set of FA synthesis parameters (relative error < 15%, RSD < 15%) whenever the fractional contribution of the tracer (*D_2_*) and the parameters to be calculated for a given FA (i.e. *S*, *E_1_*, *E_2_*, *E_3_* and *E_4_*) fall within the 0.05–0.9 range. This ensures its applicability in an actual biological scenario.

### FAMetA enables straightforward FA metabolism analyses

To evaluate FAMetA performance, a variety of *in vitro* and *in vivo* experimental settings are used. First, mouse CD8^+^ T cells are incubated for 72 h with different uniformly ^13^C labeled tracers (U-^13^C-glucose, U-^13^C-glutamine, U-^13^C-lactate, or U-^13^C-acetate) in the presence or absence of well-known inhibitors of FA metabolism enzymes [i.e. FASN (GSK2194069, FASNi) [21], SCD1 (A93572, SCD1i) [22, 23] and FADS2 (SC26196, FADS2i) [24]]. Total lipids are extracted from cell pellets and saponified to release FAs, which are subsequently analyzed by LC–MS.

**Figure 4:**
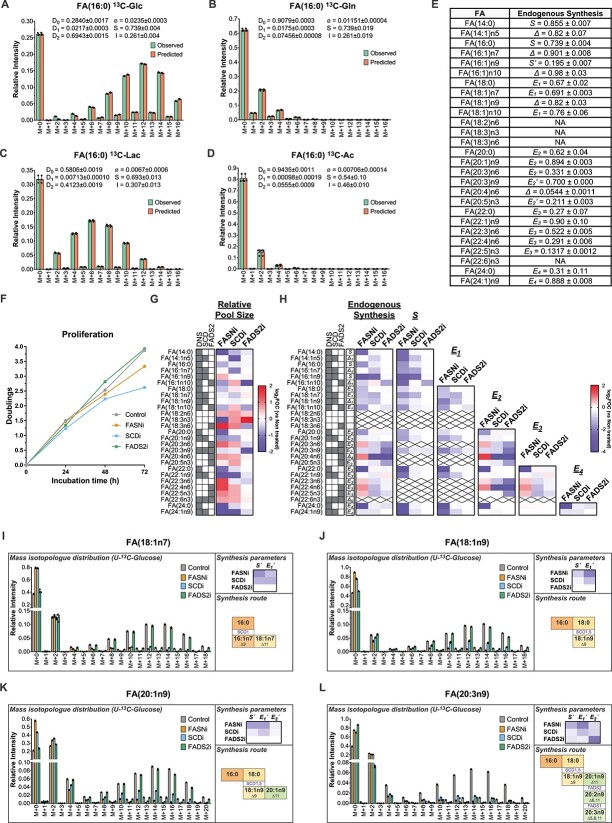
Biological validation of FAMetA in active mouse CD8^+^ T cells. Estimation of the FA metabolism parameters in the active mouse CD8^+^ T cells incubated for 72 h with various U-^13^C-tracers. (**A**–**D**) Estimation of the sources and the DNL parameters for FA(16:0) upon incubation with U-^13^C-glucose (A), U-^13^C-glutamine (B), U-^13^C-lactate (C) or U-^13^C-acetate (D). (**E**) Summary of the endogenously synthesized fraction for the 27 known FAs detected in the active mouse CD8^+^ T cells upon incubation with U-^13^-Cglucose. (**F**–**L**) Analysis of alterations in FA biosynthesis in the active mouse CD8^+^ T cells incubated for 72 h with U-^13^C-glucose induced by FASN inhibitor GSK2194069, SCD inhibitor A93572 and FADS2 inhibitor SC26196. (F) Mean proliferation of the active mouse CD8^+^ T cells during the 72-h incubation period. (G) Heatmap showing for each identified FA the mean value of the log_2_ fold-of-change (versus untreated) in the relative pool size. (H) Heatmap showing the mean value of the log_2_ fold-of-change (versus untreated) for each identified FA in the following parameters: endogenously synthesized fraction, calculated *S*, *E*_1_, *E*_2_, *E*_3_ and *E*_4_. For each FA, the parameter reported for the endogenous synthesis is indicated. (I–L) Mass isotopologue distribution, the mean value of the log_2_ fold-of-change (versus untreated) in the synthesis parameters and synthesis route for FA(18:1n7) (I), FA(18:1n9) (J), FA(20:1n9) (K) and FA(20:3n9) (L). In all cases, *n* = 3. Individual points are shown for the mass isotopologue distributions, and the mean values are reported elsewhere. The shadowed cells in (B) and (C) indicate the activities (DNS, SCD or FADS2) involved in the synthesis of a particular FA. On the heatmaps, crosses indicate missing or NA values. In (I–L), the horizontal transitions in the synthesis route description denote elongations (enzymes not indicated), and vertical transitions denote desaturations (enzymes indicated).

Twenty-seven known FAs are detected in the samples, including a variety of saturated, monounsaturated and polyunsaturated FAs within the range from 14 to 24 carbons. FAMetA accurately models the obtained mass isotopologue distributions for all of them and extracts valuable biological information about nutrient preferences and metabolic origin of each particular FA (Figure 4A–E, Supplementary Results).

Treatment with FASNi and SCD1i slightly decreases cell proliferation, but FADS2i does not (Figure 4F). Changes in the relative pool size of the detected FAs appear (Figure 4G); e.g. SCD1i lowers the intracellular levels of the n5, n7 and n9 series FAs, and increases the relative abundance of FADS2 products [e.g. sapienic acid, FA(16:1n10)], whereas FADS2i considerably diminishes sapienic acid abundance, which is consistent with previous reports on the complementary and compensatory roles of SCD1 and FADS2 [25] (Figure 4G). When analyzing endogenous synthesis, the changes reveal which enzymes are involved in the synthesis of each identified FA. FASNi decreases the endogenous synthesis of all the FAs that come from FA(16:0), and SCD1i and FADS2i decrease the endogenous synthesis of all the FAs that these enzymes are involved in (e.g. n9 series FAs for SCD1i, n10 series FAs for FADS2i) (Figure 4H). When focusing on each calculated synthesis parameter, identifying the step in which each enzyme acts and mapping synthesis routes are straightforward. For example, for FA(18:1n7) and FA(18:1n9), SCDi differentially affects synthesis parameters. In FA(18:1n9), where SCD acts at the 18-carbon level, the most prominent decrease is in calculated *E_1_* (i.e. *E_1_’* = E_1_*Δ), in FA(18:1n7), where SCD acts at the 16-carbon level, both calculated S (i.e. *S′* = S*Δ), and *E_1_* decreases upon treatment with SCDi (Figure 4I–J). The SCDi inhibition pattern observed in FA(18:1n9) is mirrored in FA(20:1n9) and FA(20:3n9) (Figure 4H–L). In addition, FADS2i decreases the calculated *E_2_* (i.e. *E_2_’* = E_2_*Δ) for FA(20:3n9), which is indicative of FADS2 introducing a double bond at the 20-carbon level (Figure 4L). Thus, FAMetA allows the identification of both changes in general patterns and particular synthesis parameters induced by FA metabolism inhibitors.

Then we move on to analyze previously published data generated using *in vitro* (H1299 cells incubated with U-^13^C-glucose and U-^13^C-glutamine, where the down-regulation of SREBP cleavage activating protein, a key protein in the regulation of FA metabolism, is induced) [14] (Supplementary Figure 10) and *in vivo* (incorporation of U-^13^C-fructose into saponified circulating FAs in wild-type and intestine-specific ketohexokinase (KHK-C) knockout mice after drinking normal water for 8 weeks, or 5 or 10% sucrose water) [26] (Supplementary Figure 11) experimental models. FAMetA properly fits the experimental FA distributions and calculates synthesis parameters for the complete array of detected FAs, and in both cases, the more detailed characterization of FA metabolism provided by FAMetA enables to decipher biological insights that were overlooked by the authors of the studies using previously available tools (please see Supplementary Results for a detailed description of the analysis of previously published data using FAMetA, including an in-depth comparison between FAMetA and FASA using the H1299 cell dataset [14]).

### FAMetA enables the identification of unknown FAs in biological samples

**Figure 5:**
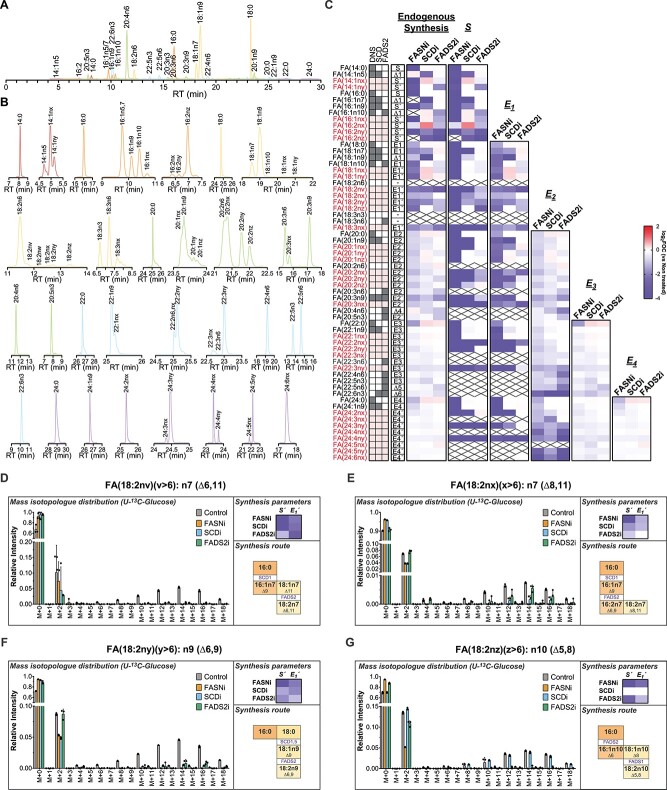
Elucidation of the synthesis route of unidentified FA species by combining FAMetA and FA metabolism inhibitors. Analysis of alterations in the FA metabolic network in the human NSCL cell line A549 incubated for 72 h with U-^13^C-glucose induced by FASN inhibitor GSK2194069, SCD inhibitor A93572, and FADS2 inhibitor SC26196. (**A**–**B**) Chromatographic separation of the saponified FAs from the A549 cells in culture. (A) Combined chromatogram showing all the detected FAs. (B) Individual chromatograms for each detected FA. (**C**) Heatmap showing the mean value of the log_2_ fold-of-change (versus untreated) for each detected FA in the following parameters: endogenously synthesized fraction, calculated *S*, *E*_1_, *E*_2_, *E*_3_ and *E*_4_. For each FA, the parameter reported for the endogenous synthesis is indicated. The shadowed cells indicate the activities (DNS, SCD or FADS2) involved in the synthesis of a particular FA. Red denotes the FAs whose synthesis route is unknown. On the heatmap, crosses indicate missing or NA values. (**D**–**G**) The mass isotopologue distribution, the mean value of the log_2_ fold-of-change (versus untreated) in the synthesis parameters and the proposed synthesis route for FAs FA(18:2nv) (D), FA(18:2nx) (E), FA(18:2ny) (F) and FA(18:2nz) (G) whose identities do not match any standard employed for the method development. In all cases, *n* = 3. Individual points are shown for the mass isotopologue distributions. The mean values are reported elsewhere. In the synthesis route description, horizontal transitions denote elongations (enzymes not indicated) and vertical transitions depict desaturations (enzymes indicated).

**Figure 6:**
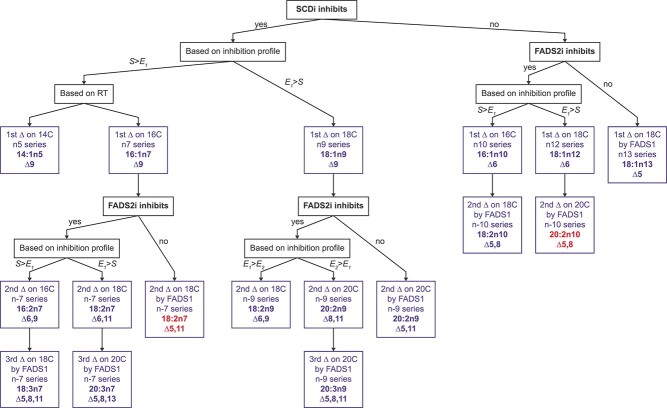
The algorithm employed to identify unknown FAs by the reconstruction of their biosynthesis route. The depicted algorithm is applied to identify the double bond positions for FAs based on the inhibition profile obtained upon incubation with U-^13^C-glucose, either with or without SCDi or FADS2i. The algorithm applies to FAs whose origin can be tracked to FA(14:0)/FA(16:0). The previous assumptions must be met: (i) the FA incorporates labeling and intensity suffices to obtain values for all/most expected isotopomers; (ii) FASNi decreases parameter S or distribution is consistent with the origin being FA(14:0)/FA(16:0). Based on the chromatographic profile, we expect the FAs to elute by increasing n-series [i.e. RT(n5 series) ≤ RT(n7 series) ≤ RT(n9 series), etc.]. The algorithm allows identifying the initial FA for the FA synthesis routes described in Figure 7; thus, the actual position of the double bonds has to be extrapolated for the FAs of a different carbon length to that indicated in the algorithm. In red, FAs for which we can anticipate the identification and synthesis route based on the described strategy, but were not detected or unambiguously assigned experimentally in the A549 cells because FADS1 inhibitors were lacking.

**Figure 7:**
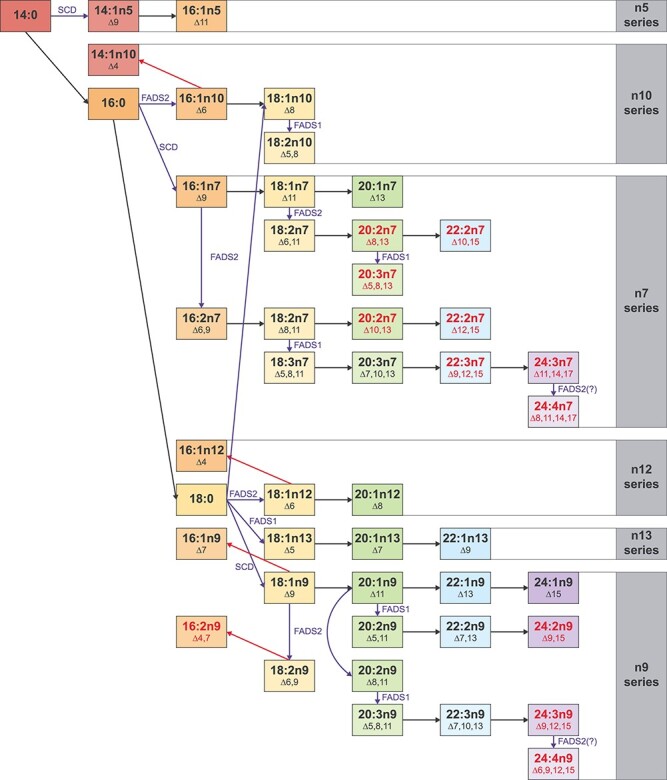
FA biosynthesis routes in the NSCLC cell line A549. Summary of the FA metabolism network in the A549 cells for those FAs that come from DNL. Black arrows denote elongations, blue arrows denote desaturations (the responsible enzyme is indicated) and a red arrow denotes degradation. Red depicts the FAs that have not been previously described.

The analysis of total FAs in the non-small cell lung cancer (NSCLC) cell line A549 reveals high FA diversity (62 species), including several FAs (33) that do not match available standards (Figure 5A–B). We hypothesize that the information provided by the retention time of each FA combined with the FAMetA analysis of the MS-data generated using U-^13^C-glucose and well-characterized inhibitors (i.e. FASNi, SCDi and FADS2i) would provide a valuable strategy to identify unknown and unexpected FAs by the reconstruction of their metabolic synthesis route. All the detected unknown FAs incorporate ^13^C from U-^13^C-glucose, which confirms their endogenous metabolic origin. In all the cases the information provided by the inhibition profile and the retention time allowed us to propose identities for them all (Figure 5C–G, Supplementary Figure 12A–AC). For example, we detect and calculate synthesis parameters for five FA(18:2) (18:2n6, nv, nx, ny, nz). Based on the decision tree depicted in Figure 6, which guides the identification of each double bond position based on the inhibition profile, we identified them as FA(18:2n7)(Δ6,11), FA(18:2n7)(Δ8,11), FA(18:2n9)(Δ6,9) and FA(18:2n10)(Δ5,8), respectively (Figure 5D–G) (please see Supplementary Results for a detailed description of the rationale behind the identification of 18:2 FAs in A549). Of the identities proposed based on the metabolic reconstruction of the biosynthesis route, 11 are confirmed with commercially available standards (Supplementary Figure 12AD–AJ), and 9 of them do not match previously described FAs (Figure 7). Thus, FAMetA and our proposed strategy disclose a more comprehensive FA biosynthetic landscape of A4594 cells, including the description of novel FAs (Figure 7).

## Discussion

The therapeutic inhibition of specific FA metabolic enzymes/transporters has been proposed in diseases like cancer [21, 23, 27–29], non-alcoholic fatty liver disease [30], autoimmunity [31] or viral infection [32]. Metabolic plasticity in FA desaturation has been recently acknowledged as a relevant phenomenon that supports lipid biosynthesis [25, 33] and confers a metabolic advantage upon SCD inhibition in cancer cells [25]. The expression of particular elongases (e.g. ELOVL2 in glioma [34] or ELOVL5 in prostate cancer [35]) supports cell growth, tumor initiation and metastasis. Despite the wide variety of FAs, their biosynthetic routes and proven functions, current state-of-the-art tools/algorithms do not provide a comprehensive characterization of FA metabolism. The most commonly used algorithm (i.e. ISA) was initially developed for the determination of DNL for FA(14:0) and FA(16:0) [11, 12]. Further developments enabled the estimation of elongations [13, 14] and of the *de novo* synthesis of odd-chain FAs [36]. Additionally, a simple strategy for the estimation of the desaturation of FA(18:1n9) based on the ratio of the total labeling of FA(18:0) and FA(18:1n9) has been also proposed [15, 19]. The shown relevance of long FAs, the importance of desaturation in cell biology and in the physiopathology of many diseases and the lack of a tool that performed a comprehensive characterization of all the biosynthetic reactions within FA metabolism in a user-friendly platform accessible to the broad lipid metabolism community have motivated us to develop FAMetA.

Our results demonstrate that FAMetA deciphers both patterns of global changes and detailed information about alterations in the synthesis route of FAs of interest both *in vitro* and *in vivo* (Figure 4, Supplementary Figures 10–11). The use of U-^13^C-glucose and well-characterized inhibitors of FA metabolism enzymes (i.e. FASN, SCD1 and FADS2), combined with FAMetA data analysis, enables the comprehensive characterization of the FA biosynthetic network in A549 cells (Figure 5, Supplementary Figure 12). Strikingly, it also discloses the identity of 12 novel FAs that belong to already described n-series, which extends the known FA biosynthesis network compared to previous tools (Figure 7). The lack of well-characterized inhibitors of FADS1 or elongases (ELOVL1-7) limits the level of detail that can be achieved when identifying FAs by their metabolic reconstruction. Likely, some detected FAs, which are identified as the product of double desaturation introduced by the consecutive action of SCD1 and FADS2, are instead a mixture in which the products of a double desaturation introduced by SCD1 and FADS1 are also present. So the unambiguous identification of the proposed unknown/novel FAs would require using complementary analytical tools and, if possible, authentic chemical standards. Nevertheless, we demonstrate that FAMetA enables the straightforward mapping of FA biosynthetic pathways by the techniques and reagents routinely used in metabolism studies.

Compared to previous tools FAMetA offers (Supplementary Table 1): (i) the characterization of a broader FA biosynthesis network as it includes in a single tool DNL, elongation and desaturation; (ii) the possibility of running the required steps from data preprocessing to analysis of FA metabolism and graphical representation in a single tool; (iii) a user-friendly environment thanks to its implementation as an R package and a web-based app; (iv) better fitting to the experimental data thanks to the implementation of quasi-multinomial fitting that incudes the parameter *Φ* that accounts for data overdispersion; (v) better description of elongations, thus enabling an easier interpretation of the estimated parameters; (vi) easy-to-interpret parameters and graphical representations that lead to obtain meaningful biological conclusions.

Future developments of mass isotopologue data analysis tools, including FAMetA, should address some unresolved issues like the use of labeled-FAs as nutrients, distinguishing the uptake of exogenous FA and the lipolysis of stored lipids, estimating the synthesis rate of the FAs that result from the degradation of a longer FA [e.g. FA(16:1n9), where *S′* = S*E_1_*degradation], or the resolution of the FA metabolism properties of particular lipid classes of interest or organelles. Additionally, the FAMetA algorithm is exclusively designed to fit the data from ^13^C-based tracers for even-chain FAs. Thus, future efforts should focus on implementing calculations based on ^2^H-tracers, such as ^2^H_2_O, which contributes to FA synthesis via direct H_2_O incorporation, and also via NADPH [7, 8], and to expand the reactions to cover odd-chain FAs, in which not only the lipogenic Acetyl-CoA has to be estimated, but also the lipogenic Propionyl-CoA pool [36]. The calculations based on ^2^H-tracers can be performed using the code by Zhang et al. [7], the calculations for odd-chain FAs can be performed using the code by Crown et al. [36] and the equations to model FA degradation or the use of labeled-FAs as nutrients could be theoretically implemented within software designed for the ^13^C-metabolic flux analysis such as METRAN [37], INCA [38], or 13CFLUX2 [39]. Additionally, recent developments in the field of proteomics such as the use of ^12^C-nutrients as light isotopic tracers [40], or the resolution of the isotope incorporation based on the number of labeling sites and the label enrichment using numerical techniques [41] could be implemented to estimate FA metabolism using isotopic tracers. Finally, the analysis of FA metabolism at a compartmental, lipid class, or single lipid level would require the use of complex fractionation of lipid extracts or the developments of new tools to deal with the complex distributions that arise from the labeling of each structural component of a complex lipid.

Despite these limitations, FAMetA constitutes the first tool that enables reliable estimations of FA import, synthesis, elongation and desaturation for the whole FA metabolic network of FAs within the range from 14 to 26 carbons. The FAMetA workflow includes the required functionalities (data preprocessing, FA metabolism analysis, group-based comparisons and graphical representation) to run a complete data analysis on a single platform (Figure 1). Its combination with the systematic genetic manipulation of enzymes/transporters involved in FA metabolism can contribute to the characterization of FA metabolism in unprecedented detail. Finally, to spread its use, FAMetA is freely available as an open-source R package and a web-based application (www.fameta.es). In conclusion, we believe that FAMetA is a valuable addition to existing tools and has the potential to become a key resource to study the complex FA biosynthetic landscape.

## Materials and methods

### FAMetA

#### FAMetA implementation

FAMetA was developed in an R programming environment. It is available via CRAN (https://CRAN.R-project.org/package=FAMetA). In addition, the web-based implementation of FAMetA was built using the Shiny R package (Shiny: Web Application Framework for R. 2021). It is accessible at www.fameta.es.

#### The FAMetA workflow

The FAMetA workflow starts with raw MS data files in the mzXML format, which can be obtained with any MS file converter, e.g. msConvert from ProteoWizard [42], and a csv file containing the required metadata (sample name, acquisition mode, sample group or class, and any additional information like external measures for normalization) (Supplementary Figure 2, steps 1–2). Data preprocessing can be performed in the R environment/web-based application using our proposed workflow, which combines functions from FAMetA and our previously described R-package LipidMS [43, 44] (available via CRAN (https://CRAN.R-project.org/package=LipidMS)) (Supplementary Figure 2, steps 2–5). LipidMS is called for the first preprocessing step, which runs peak-picking, alignment and grouping through functions *batchdataProcessing*, *alignmsbatch* and *groupmsbatch* (Supplementary Figure 2, step 2). Then FAMetA is called, and functions *annotateFA* and *curateFAannotations* are used to identify any unique FA isomers. Automatic FA annotations can be exported to a csv file and be modified by removing rows of unwanted FA by modifying the initial and end retention times, or by adding new rows with missing compounds. Unique compound names with nomenclature ‘FA(16:1)n7’, where n7 (omega-7) indicates the last double-bond position, are required to differentiate FA isomers. For any unknown positions, letters x, y and z are allowed (i.e. FA(16:1)nx). The internal standards for later normalization can also be added in a new row at this point by indicating IS in the compound name column (Supplementary Figure 2, step 3). Once FAs have been correctly identified, FA isotopes can be extracted using function *searchFAisotopes* (Supplementary Figure 2, step 4). Finally, data can be corrected and normalized using the *dataCorrection* function, which runs four different steps (all of which are optional): data correction for natural ^13^C abundance using the *accucor* algorithm [45]; data normalization with internal standards; blank subtraction; external normalization (Supplementary Figure 2, step 5). Alternatively, the external data processed by other available software/tools (e.g. data preprocessing using tools as ElMa) can be loaded at this workflow point or before the data correction and normalization steps.

Then the actual FA metabolism analysis can be performed by sequentially running the *synthesisAnalysis*, *elongationAnalysis* and *desaturationAnalysis* functions (Supplementary Figure 3, steps 1–3). The first two functions model isotopologue distributions by non-linear regression (https://CRAN.R-project.org/package=minpack.lm) with many initial values [46] to ensure that the best fits are found. By default, a maximum of 1,000 iterations for synthesis and 10,000 for elongation are performed for each set of initial values to fit the isotopologue distributions (*maxiter* parameter) or until the model has converged 100 times (*maxconvergence* parameter). If no results are obtained or parameters come close to the limits of the confidence intervals, these parameters can be increased to improve the results. The third function employs the previous results to estimate the desaturation values. Finally, the summarized results tables and heatmaps are obtained using the *summarizeResults* function to export and explore the results (Supplementary Figure 3, step 4).

#### Model assumptions

(i) The acetyl-CoA pool contributing to lipogenesis has a uniform labeling pattern. (ii) The lipogenic acetyl-CoA pool reaches isotopic steady state quickly compared with the total labeling time. (iii) For FAs of 16 or more carbons the final product of FASN is FA(16:0). (iv) For the FAs belonging to the n3 and n6 series, *S* = 0. (v) At any given time point *I* = import + pre-existing FAs, and only when the pre-existing FAs have been completely replaced (the actual steady state has been achieved) *I* = import. (vi) There is a single FA pool. (vii) The data have been corrected to account for the natural abundance of the ^13^C isotopes.

#### Data requirements for FA modeling

Before FA metabolism analysis, the user should check that the FAs of interest have been labeled enough to obtain isotopologue distributions of good quality (avoid missing isotopologues) that guarantee the calculated parameters fall within the ranges that allow their accurate estimation. When curating FA annotations, FA names must follow the nomenclature FA(C:d)ns, where C is the total number of carbon, d is the number of unsaturations and ns refers to the omega series, which indicates the position of the last double bound starting from the end of the chain. Duplicated identities are not allowed and the series must belong either to known series [i.e. 3, 5, 6, 7, 7a (i.e. second double bond introduced by FADS2 at 16C), 7b (i.e. second double bond introduced by FADS2 at 18C), 9, 10, 12, 13], or use the letters x, y and z for an unknown series. For the estimation of synthesis parameters, data must have been corrected to account for the natural abundance of the ^13^C isotopes.

#### Estimation of the DNL parameters

We considered FA(16:0) the final DNL product. Thus, FAMetA can estimate the DNL parameters for FAs up to 16 carbons. For these species, *I* and *S* represent the fraction of the FA pool that is imported and synthesized, respectively, and sums 1:

(1) \begin{equation*} {I}_{16:0}+{S}_{16:0}=1 \end{equation*}

For the DNL analysis, FA isotopologue distributions (previously corrected for the natural abundance of the ^13^C isotopes) are modeled with the following sum of the weighted quasi-multinomial distributions adapted from [47]:

(2) \begin{equation*} P\left(m=0\right)=I+S\ast \left(1+N\ast \varPhi \right)\ast \frac{\ {D}_0}{1+N\ast \varPhi}\ast{\left(\frac{D_0+N\ast \varPhi }{1+N\ast \varPhi}\right)}^{N-1} \end{equation*}

(3) \begin{equation*} P(m)=\sum_{j=1}^kP\left({X}_0={x}_{0,j},{X}_1={x}_{1,j},{X}_2={x}_{2,j}\right);\mathrm{for}\ 1\le m\le M \end{equation*}

where

(4) \begin{align*} &P\left({X}_0={x}_{0,j},{X}_1={x}_{1,j},{X}_2={x}_{2,j}\right)\nonumber\\\nonumber&=S\ast \frac{N!}{x_{0,j}!{x}_{1,j}!{x}_{2,j}!}\ast \left(1+N\ast \varPhi \right)\ast \frac{D_0}{1+N\ast \varPhi}\ast{\left(\frac{D_0+{x}_{0,j}\ast \varPhi }{1+N\ast \varPhi}\right)}^{x_{0,j}-1}\\ &\ast \frac{D_1}{1+N\ast \varPhi}\ast{\left(\frac{D_1+{x}_{1,j}\ast \varPhi }{1+N\ast \varPhi}\right)}^{x_{1,j}-1}\!\ast \frac{D_2}{1+N\ast \varPhi}\ast{\left(\frac{D_2+{x}_{2,j}\ast \varPhi }{1+N\ast \varPhi}\right)}^{x_{2,j}-1} \end{align*}

given that

(5) \begin{equation*} {\displaystyle \begin{array}{c}{x}_{i,j}=0,1,\dots, N\\{}\sum_{i=1}^2{x}_{i,j}={x}_{0,j}+{x}_{1,j}+{x}_{2,j}=N\end{array}} \end{equation*}

(6) \begin{equation*} \sum_{i=1}^2i\ast{x}_{i,j}=0\ast{x}_{0,j}+1\ast{x}_{1,j}+2\ast{x}_{2,j}=m \end{equation*}

(7) \begin{equation*} 0\le \varPhi \le \frac{1-\max \left({D}_0,{D}_1,{D}_2\right)}{N} \end{equation*}

*M* is the total number of carbons in the FA molecule and *N* = *M*/2. This represents the number of acetyl-CoA molecules used for the synthesis of an FA of length *M*. *m* is the number of ^13^C atoms incorporated into the FA molecule. *D_0_*, *D_1_* and *D_2_* represent the fraction of acetyl-CoA with 0, 1, or 2 atoms of ^13^C, respectively, and sum 1. *x_0_*, *x_1_* and *x_2_* represent the number of acetyl-CoA units with 0, 1, or 2 ^13^C atoms that provide an M-carbon FA with an m label. For a given pair of *N* and *m* values, up to *k* combinations of the *x_0_*, *x_1_* and *x_2_* values fulfill equations (5) and (6). *Φ* accounts for overdispersion and can be set at 0 to reduce quasi-multinomial distribution to multinomial distribution. The *in silico* validation of the above-described equations demonstrates an overestimation of *Φ* and an underestimation of *S* and *D_2_* for values of *D_2_* ≥ 0.75. In these situations, the upper limit of *Φ* is set at 0.5*(1 − max(*D_0_*, *D_1_*, *D_2_*)/*N*). Note that overdispersion parameter *Φ* modifies *D_0_*, *D_1_* and *D_2_* for each synthesis step, which allows distribution to widen or narrow.

Based on this model, non-linear regression (https://CRAN.R-project.org/package=minpack.lm) with many sets of plausible initial values (adapted from ref [46]) is used to fit the observed isotopologue distributions of FAs up to 16 carbons, and to estimate parameters *D_1_*, *D_2_*, *Φ* and *S*. When analyzing multiple samples per group, *S* and *D_2_* values can be checked to ensure homogeneity within each group. If not, we can assume *D_2_* should remain within a narrow range for a given condition and thus fix *D_2_* by the mean of the rest of the samples in the group for the outlier sample and repeat the analysis to improve the calculation of the *S* value. To improve the analysis results, the *D_1_*, *D_2_* and *Φ* values obtained for FAs up to 16C are used to model the distribution of FAs of 18-to-26C.

#### Elongation

The main product of the DNL of FA is FA(16:0) [1]. Therefore, the main DNL route, plus elongation, starts at 16 carbons and then adds blocks of two carbons. Elongation from FA(14:0) is a minor route [14] and is omitted for simplicity. For the FAs ranging from 18 to 26 carbons, the following equations are considered:

(8) \begin{equation*} {I}_{18:0}+{E}_1\left({I}_{16:0}+{S}_{16:0}\right)={I}_{18:0}+{E}_1=1 \end{equation*}

(9) \begin{equation*} {I}_{20:0}+{E}_2\ast \left({I}_{18:0}+{E}_1\ast \left({I}_{16:0}+{S}_{16:0}\right)\right)={I}_{20:0}+{E}_2=1 \end{equation*}

(10) \begin{equation*} {I}_{22:0}+{E}_3\ast \left({I}_{20:0}+{E}_2\ast \left({I}_{18:0}+{E}_1\ast \left({I}_{16:0}+{S}_{16:0}\right)\right)\right)={I}_{22:0}+{E}_3=1 \end{equation*}

(11) \begin{align*} {I}_{24:0}+{E}_4 &\ast \left({I}_{22:0}+{E}_3\ast \left({I}_{20:0}+{E}_2\ast \left({I}_{18:0}+{E}_1\ast \left({I}_{16:0}+{S}_{16:0}\right)\right)\right)\right)\nonumber\\ &={I}_{24:0}+{E}_4=1 \end{align*}

(12) \begin{align*} {I}_{26:0}+{E}_5 &\ast ({I}_{24:0}+{E}_4\ast ({I}_{22:0}+{E}_3\ast ({I}_{20:0}+{E}_2\ast ({I}_{18:0}+{E}_1 \nonumber\\ &\ast ({I}_{16:0}+{S}_{16:0})))))={I}_{26:0}+{E}_5=1 \end{align*}

For the elongation analysis of endogenous FA, isotopologue distributions are modeled using equation (2) for synthesis until FA(16:0), followed by single independent elongation steps (*E_1_*, *E_2_* …, *E_n_*). The probability of incorporating 0, 1, or 2 ^13^C atoms into the FA to be elongated equals *E_i_D_0_*, *E_i_D_1_* and *E_i_D_2_*, respectively. For FA longer than 16C, only synthesis and elongation terms are estimated (*S*, *E_1_*, *E_2_* …, *E_n_*), whereas the rest (*D_0_*, *D_1_*, *D_2_* and *Φ*) are inherited from the results obtained for the FA(16:0). In case no results are available for FA(16:0), FAMetA uses FA(14:0), mean of all FA of 16C (FA(16:X)), or mean of all FA of 14C (FA(14:X)) in this order of priority. For FA(18:0), FA isotopologue distributions (previously corrected for natural ^13^C isotopes abundance) are modeled with the following equations:

(13) \begin{equation*} {P}_{18:0}\left(m=0\right)={I}_{18:0}+{E}_1\ast{D}_0\ast{P}_{16:0}\left(m=0\right) \end{equation*}

(14) \begin{equation*} {P}_{18:0}\left(m=1\right)={E}_1\ast{D}_0\ast{P}_{16:0}\left(m=1\right)+{E}_1\ast{D}_1\ast{P}_{16:0}\left(m=0\right) \end{equation*}

(15) \begin{equation*} {\displaystyle \begin{array}{c}{P}_{18:0}(m)={E}_1\ast{D}_0\ast{P}_{16:0}\left(m=m\right)+{E}_1\ast{D}_1\ast{P}_{16:0}\left(m=m-1\right)+{E}_1\\\ast{D}_2\ast{P}_{16:0}\left(m=m-2\right) {}\mathrm{for}\ 2\le m\le M;{P}_{16:0}\left(m>16\right)=0\end{array}} \end{equation*}

Analogous equations can be obtained for FA with *M* > 18 by adding elongation terms to previously existing distributions. For series n6 and n3 (Supplementary Figure 1), elongation is usually expected from FA(18:2)n6 and FA(18:3)n3. Thus, synthesis (*S*) and the first elongation step (*E_1_*) are set at 0. If isotopologue *M* + 2 is observed, given the degradation of FA(18:2)n6 or FA(18:3)n3, followed by one elongation step, then *E_1_* is estimated. However, the endogenously synthesized fraction remains at NA. In addition, isotopologue distributions of FA longer than 16C are checked to decide if any parameter can be fixed to 0 (for those parameters selected based on the omega series). At least two or three even isotopologues (*M* + 2, *M* + 4, *M* + 6, …), with a relative intensity greater than 0.1 or 0.01%, respectively, along the whole distribution, are required to estimate *S*. Similarly, for elongation terms, specific isotopologues are checked to ensure how many elongation steps have occurred (*M* + *x* > 0.1%). Once again, non-linear regression (https://CRAN.R-project.org/package=minpack.lm) with multiple initial values [46] is used to fit the observed isotopologue distributions of the elongated FAs.

#### Desaturation

After estimating the synthesis and elongation parameters, these results can be used to calculate the FA fraction that comes from desaturation in the unsaturated FA. For a given unsaturated FA (e.g. FA(18:1n9)), we can conceptually consider a one-step elongation-desaturation reaction (in this example, directly from FA(16:0) to FA(18:1n9)), or a two-step elongation followed by a desaturation process (in this example, FA(16:0) is elongated to FA(18:0) and then desaturated to FA(18:1n9)) (Figure 3). By using FAMetA, we can directly estimate both *E_1_* and *E_1_’* from the isotopologue distributions of FA(18:0) and FA(18:1n9), respectively. From alternative paths, the relative import and endogenous synthesis pathways of FA(18:1n9) can be written as

(16) \begin{equation*} {I_{18:1n9}}^{\prime }+{E_1}^{\prime}\ast \left({S}_{16:0}+{I}_{16:0}\right)=1 \end{equation*}

(17) \begin{equation*} {I}_{18:1n9}+\varDelta \ast{E}_1\ast \left({S}_{16:0}+{I}_{16:0}\right)+\varDelta \ast{I}_{18:0}=1 \end{equation*}

By combining both equations, we can define that

(18) \begin{equation*} {I_{18:1n9}}^{\prime }={I}_{18:0}\ast \varDelta +{I}_{18:1n9} \end{equation*}

and, thus, calculate desaturation parameter Δ as

(19) \begin{equation*} \varDelta =\frac{{E_1}^{\prime }}{E_1} \end{equation*}

If both *E_i_’* and *E_i_* are below the confidence interval, which is set at 0.05, by default, for desaturation, parameter Δ is not calculated, and *E_i_’* remains as the endogenously synthesized fraction. If the stationary state is not reached, values >1 can be obtained for the desaturation parameter, that is, in this case, replaced with 1.

This same model can be used for all the known desaturation steps, provided that the precursor and product FA isomers are correctly and uniquely identified, and the stationary state is reached. For the FA synthesized from desaturation activities, Δ is considered the fraction from endogenous synthesis. So the imported fraction is calculated as 1 − Δ. With unknown isomers or missing precursors, *S′* or *E’* is returned for the DNS of FAs until 16 carbons or the elongation of longer FAs, respectively. The reactions included in FAMetA are described in Supplementary Figure 1 [14, 16, 48, 49]. However, additional reactions (desaturations) can be included for unknown/additional FAs by modifying *desaturationdb* in FAMetA.

#### In silico tests of FAMetA

To test FAMetA’s performance with different FA isotopologue distributions and noise levels, *in silico* tests on models are run. To evaluate FAMetA’s performance to estimate parameters for the DNS analysis, realistic values for *D_1_* (5 values from 0 to 0.2), *D_2_* (15 values from 0 to 1), *Φ* (10 values from 0 to 0.1) and *S* (15 values from 0 to 1) are combined to simulate 3,945 theoretical FA(16:0) distributions to which 0, 2, 5 and 10% noise levels are added to obtain 10 different noised distributions for each set of parameters. Bias (evaluated as an absolute or relative error) and dispersion (evaluated as RSD) are calculated and graphically represented for parameters *D_2_*, *S* and *Φ* (Supplementary Figure 4).

To evaluate FAMetA’s performance to estimate the parameters for the elongation analysis, the mass isotopologue distributions for FA(18:0), FA(20:0), FA(22:0) and FA(24:0) are generated. To evaluate the elongation of FA(16:0) to FA(18:0), *D_1_* and *Φ* are set at 0.05 and 0.01, respectively. The realistic values for *D_2_* (9 values between 0.1 and 0.9), *S* (19 values between 0.05 and 1 and *E_1_* (19 values between 0.05 and 1) are employed to generate 3,249 theoretical FA(18:0) distributions. For FA(20:0), FA(22:0) and FA(24:0), the synthesis parameters for FA(16:0) are set at *D_1_* = 0.05, *Φ* = 0.01 and *S* = 0.6. Nine values within the 0.1–0.9 range and 10 values within the 0.1–1 range are generated for *D_2_* and *E_n_*, respectively. Bias (evaluated as a relative error) and dispersion (evaluated as RSD) are calculated and graphically represented for all the estimated parameters (Supplementary Figures 5–8).

To evaluate FAMetA’s performance to estimate the parameters for the desaturation analysis, the mass isotopologue distributions for FA(16:1n7) and FA(18:1n9) are generated. *D_1_* and *Φ* are set at 0.05 and 0.01, respectively. For FA(16:1n7), 13 values within the 0.1–0.87 range and 14 values within the 0.07–1 range are generated for *D_2_* and *S*, respectively. For FA(18:1n8), 13 values within the 0.1–0.87 range and 14 values within the 0.07–1 range are generated for *D_2_* and *E_1_*, respectively. In both cases, 14 values within the 0.07–1 range are generated for Δ. Bias (evaluated as a relative error) and dispersion (evaluated as RSD) are calculated and graphically represented for parameter Δ for both FAs (Supplementary Figure 9).

### Reagents, biological sources and experimental details

Detailed description of reagents, cell isolation and culture, animal models, cell lines and methods to extract and analyze FAs is provided in the Supplementary Information.

**Key Points**

- Stable isotope tracers can be used to study FA metabolism.
- Current tools focus on DNL and FASA recently incorporated estimation of elongation.
- FAMetA improves the determination of DNL and elongation and implements the systematic estimation of desaturation.
- FAMetA outperforms previous tools and enables the straightforward analysis of alterations in FA metabolism.
- FAMetA provides all the functionalities needed for the complete analysis of MS-based FA isotopologue data in a freely available R package and a friendly web-based application.

## Supplementary Material

Supplementary_Information_020123_Briefings_in_Biooinformatics_bbad064

## Data and materials availability

The input mzXML LC–MS data files used to generate Figures 2–4, the metadata associated with the studies, the FA identities for each dataset, the preprocessed data ready to be used to estimate the FA metabolism parameters for each study and an R script are available at Zenodo with accession number 6511248. FAMetA’s source code is offered to the public as a freely accessible software package under the GNU GPL license, version 3. It is available at https://github.com/maialba3/FAMetA and Zenodo with accession number 6511248. FAMetA R package available at CRAN (https://CRAN.R-project.org/package=FAMetA) will be maintained at least for the following 5 years.
