# Supplementary material for: FAMetA: a mass isotopologue-based tool for the comprehensive analysis of fatty acid metabolism
Source: Brief Bioinform. 2023 Mar 1;24(2):bbad064. doi: 10.1093/bib/bbad064 (PMC10025582; doi:10.1093/bib/bbad064)
Supplement: Supplementary_Information_020123_Briefings_in_Biooinformatics_bbad064 [file supplementary_information_020123_briefings_in_biooinformatics_bbad064.docx]

Supplementary Materials for

FAMetA: a mass isotopologue-based tool for the comprehensive analysis of fatty acid metabolism

María I. Alcoriza-Balaguer ^1,^†, Juan C. García-Cañaveras ^1,^†, Marta Benet ^1^, Oscar Juan Vidal^1^ and Agustín Lahoz ^1,2,*^

^1^ Biomarkers and Precision Medicine Unit, Medical Research Institute-Hospital La Fe, Av. Fernando Abril Martorell 106, Valencia, 46026, Spain.

^2^ Analytical Unit, Medical Research Institute-Hospital La Fe, Av. Fernando Abril Martorell 106, Valencia, 46026, Spain.

^†^ These authors contributed equally.

^*^ Corresponding author: agustin.lahoz@uv.es

**This PDF file includes:**

Supplementary Results

Supplementary Materials and Methods

Supplementary Figures 1 to 11

Supplementary Table 1

Supplementary Results

**Nutrient preferences and FA sources in mouse effector CD8^+^ T cells.**

Under standard culture conditions (i.e., RPMI media and normoxia), glucose is the preferred carbon source for FA synthesis (D ≈ 0.7) in the active mouse CD8^+^ T-cells (**Figure 4A**), with a minor contribution of glutamine (D ≈ 0.08) (**Figure 4B**). If present in media, lactate and glucose are metabolically exchangeable at the lactate dehydrogenase (LDH) level. Thus, lactate feeds the pyruvate pool and FA synthesis (**Figure 4C**). When supplemented in media, acetate feeds the acetyl-CoA pool and contributes to FA synthesis (**Figure 4D**). These results are consistent with previously published data obtained by different approaches [1–3]. Although most of the identified FAs appear in culture media, endogenous synthesis is the preferential route for the saturated and monounsaturated FAs, whereas polyunsaturated FAs preferentially come from exogenous sources (**Figure 4E**).

**Comparison between FAMetA and FASA**

The comparison between FAMetA and FASA is performed using a dataset published by the authors of FASA consisting of eight samples and twelve FAs in the H1229 cells incubated with U-^13^C-glucose and U-^13^C-glutamine either with or without the induced down-regulation of the SREBP cleavage activating protein (SCAP), a key protein in the regulation of FA metabolism, is induced (shControl or shSCAP) [4]

We firstly compare them in computing speed terms. The processing time with Intel Xeon E5-1620 CPU (3.5 GHz) with 32 GB RAM in Windows is ~170 min for FASA (Matlab R2022a) and ~ 12 min for the FAMetA R package (R v4.1.1, RStudio v1.4, FAMetA v0.1.3). The same analysis on the FAMetA webserver takes ~30 minutes. The FAMetA algorithm calculates the fractional contribution of the carbon source (*D_0_*, *D_1_* and *D_2_*) and overdispersion parameter (*Φ*) based on the distribution of FA(16:0). These values are then employed to fit the remaining FAs. The same strategy is employed for FASA by firstly fitting FA(16:0) and then the remaining FAs by setting the *D_0_*, *D_1_* and *D_2_* values.

FAMetA and FASA present differences in the way they calculate the FA biosynthesis parameters. While FAMetA calculates import, DNL, elongation and desaturation, FASA does not calculate desaturation. FAMetA and FASA calculate elongation by very different approaches. FAMetA provides the direct estimation of each step in a specific FA synthesis pathway (**Figure 3**). For example, the FA(20:0) sources are described as *I*_20:0_ + *E_2_* = *I*_20:0_ + *E_2_* * (*I*_18:0_ + *E_1_* * (*I*_16:0_ + *S*_16:0_)), where each parameter (*S*, *E_1_*, *E_2_*) directly represents a single synthesis route step, and *E_2_* is the direct estimation of the fraction of FA(20:0) that results from the elongation of the total FA(18:0) pool. Conversely in FASA FA(20:0), sources are described as *S* + *IE_2_* + *IE_1_* + *I*, where *S* (elongated from FA(16:0)) actually represents *S_16:0_* * *E_1_* * *E_2_*; *IE_2_* (elongated from the imported FA(16:0)), *I*_16:0_ * *E_1_* * *E_2_*; *IE_1_* (elongated from FA(18:0)), *I*_18:0_ * *E_2_*, and *I* represents the fraction of the directly imported FA(20:0) [4].

Using FASA, the authors of the original study conclude that SCAP down-regulation decreases both DNL and elongation [4]. Although they do not report the detailed results of each synthesis parameter for every reported FA, we analysed the dataset using FASA to find that several parameters change for each FA, and it is difficult to ascertain clear patterns to provide a more detailed conclusion than that proposed by the authors. Using FAMetA, we identify that SCAP down-regulation decreases the synthesis of monounsaturated n7 [i.e. FA(16:1n7) and (FA(18:1n7)] and n9 [i.e. FA(18:1n9), FA(20:1n9), and FA(22:1n9)] FAs (**Supplementary Figure 10B**). When focusing on particular synthesis parameters, the calculated *S* (i.e. *S’*= *S*Δ*) is the most altered parameter for the n7 series, which is consistent with SCD1 introducing the double bond at the 16-carbon level. The calculated *E_1_* (i.e. *E_1_’*= *E_1_*Δ*) is the most altered parameter for the n9 series, and is consistent with SCD1 introducing the double bond at the 18-carbon level (**Supplementary Figure 10B**). Our refined analysis, which includes the calculation of desaturation and easy-to-interpret direct estimations of each elongation step, identifies that the main decrease occurs in the endogenous synthesis of the SCD1-derived n7 and n9 series of FAs. This indicates diminished SCD1 activity as the main metabolic change induced after SCAP silencing.

Thus we conclude that compared to FASA, FAMetA provides a more comprehensive characterisation of the FA biosynthetic network, a better and more intuitive description of each synthesis parameter, and a more complete workflow that goes from data pre-processing to group-based comparisons and graphical representation. It is also more efficient from a computing perspective.

**FAMetA enables the analysis of FA metabolism *in vivo***

We analyze previously published data on the incorporation of U-^13^C-fructose into saponified circulating FAs in wild-type and intestine-specific ketohexokinase (KHK-C) knockout mice after drinking normal water for 8 weeks, or 5% or 10% sucrose water [5]. FAMetA properly fits the *in vivo* generated data, characterized by low synthesis, slight contribution and a high proportion of odd-labelled isotopologues, but within the high-confidence ranges established using the *in silico* validation datasets (**Supplementary Figure 11A-D**). The observed general trend suggests increased DNL, elongation and desaturation upon sucrose treatment, with a more pronounced effect on the KHK-C knockout mice (**Supplementary Figure 11E**). Neither sucrose nor KHK-C ablation influences the fractional contribution of fructose to DNL (**Supplementary Figure 11F-G**), but exposure to drinking fructose significantly alters DNL (*S*), elongation (*E_1_* and *E_2_*) and desaturation (**Supplementary Figure 11H-K**). The *post hoc* comparisons reveal significantly heightened FA(16:0) synthesis when drinking more fructose, but only in the KHK-C knockout group (**Supplementary Figure 11H**), as well as augmented desaturation when drinking more fructose in both the wild-type and KHK-C knockout mice (**Supplementary Figure 11K**). These results agree with and extend those reported by the authors of the study which reported increased total ^13^C-labelled carbons in saponified circulating palmitate that accounts for the cumulative effect of DNL, the contribution of fructose to DNL and the palmitate concentration [5].

**Detailed description of the rationale behind the identification of 18:2 FAs in A549 cells.**

Five FA(18:2) (18:2n6, nv, nx, ny, nz) are detected in the NSCLC cell line A549 (**Figure 5B**). Based on their retention time and expected n-series, v, x, y and z should be > 6 (**Figure 5B**). FA(18:2nz) is identified as FA(18:2n10) because SCDi does not affect any synthesis parameter and FADS2 decreases the calculated *E_1_* (i.e. *E_1_’* = *E_1_***Δ*) (**Figure 5G**). For FA(18:2nv) and FA(18:2nx), SCD1i decreases the calculated *S* more than *E_1_*, but the opposite occurs for FA(18:2ny). Thus FA(18:2nv, nx) and FA(18:2ny) are respectively identified as FA(18:2n7) and FA(18:2n9) (**Figure 5D-F**). Based on the FADS2i inhibition profile, we conclude that FADS2 introduces the second double bond at the 18-carbon level for FA(18:2nv) because FADS2i decreases the calculated *E_1_* more than the calculated *S*, and at the 16-carbon level for FA(18:2nx) because FADS2i decreases the calculated *S*. Therefore, the four unknown FA(18:2) are identified as FA(18:2n7)(Δ6,11), FA(18:2n7)(Δ8,11), FA(18:2n9)(Δ6,9) and FA(18:2n10)(Δ5,8), respectively (**Figure 5D-G**).

**Troubleshooting**

*1) FAMetA could not fit the data*

Increment the number of iterations and the number of initial values. This will result in an increase in processing time, but will increase the probability of finding a plausible solution.

However, it is very likely that for low quality data (i.e. extremely low labelling, incomplete isotopologue distributions…) FAMetA could be unable to fit the experimental distribution or the confidence of the reported values is not sufficient to obtain valid biological conclusions.

Some solutions, at the experimental design level include: increase the labeling time, instead of using a single labeled nutrient (e.g. glucose) use a combination of labeled nutrients to increase the *D_2_* value (e.g. glucose + glutamine + lactate), if a treatment with an inhibitor has been used, decrease the dose so that the alteration is evident, but the inhibition is not complete, increase the acquisition/accumulation time of the mass spectrometer so that very low abundant isotologues can be detected, increase the mass tolerance for data pre-processing to find missing isotopologues due to high mass error…

*2) Upon a treatment (e.g. inhibitor or genetic modification of a FA synthesis, elongation or desaturation enzyme) the reported values for S and/or D_2_ are not consistent among replicates and/or fall outside the recommended values (i.e <0.05 or >0.95).*

If there is a control condition, the *D_1_* and *D_2_* values can be fixed based on the results obtained for the control/untreated samples, assuming that the treatment does not affect nutrient preferences. Additionally the number of iterations can be also increased.

*3) FAMetA does not perform the analysis because of an error in the format of the input data.*

When curating FA annotations, FA names must follow the nomenclature FA(C:d)ns, where C is the total number of carbon, d is the number of unsaturations and ns refers to the omega series, which indicates the position of the last double bound starting from the end of the chain. Duplicated identities are not allowed and the series must belong either to known series [i.e. 3, 5, 6, 7, 7a (i.e. second double bond introduced by FADS2 at 16C), 7b (i.e. second double bond introduced by FADS2 at 18C), 9, 10, 12, 13) or use the letters x, y and z for unknown series. The database can be modified to include new FA series.

*4) The reported values for some desaturations are 1.*

Abnormally high values for desaturations can be obtained when the system is not in steady state or alternatively when the separation of the FAs is not good enough to separate all the possible isomers. In that case, the miss assignation of the FA identity can result in incorrect desaturation values. For desaturation values of ≥ 1, FAMetA returns a value of *Δ* = 1 although a warning appears.

*5) Upon a treatment (e.g. inhibitor or genetic modification of a FA synthesis, elongation or desaturation enzyme) many of the reported desaturations are NA.*

FAMetA only calculates desaturations when at least one of the synthesis parameters [e.g. either the precursor FA (*S* or *E_n_*), the product FA (*S’* or *E_n_’*) or both] are ≥ 0.05. If both of them are < 0.05 FAMetA will return a *NA* value. The user can modify that threshold calculate *Δ* when both *S* or *E_n_* and *S’* or *E_n_’* are < 0.05.

*6) The set of FAs is incomplete, how does it affect to the reported values?*

In case some of the missing FAs contain critical information for the estimation of desaturation values, desaturations for those FAs will not be calculated and FAMetA will report the synthesis parameters *S’* and/or *E_n_’*. Even in that case by comparing the results obtained between conditions/treatments, one can get useful information about alterations in desaturation activities

Supplementary Materials and Methods

**Reagents**

Standard chemical reagents were obtained from Sigma-Aldrich. RPMI 1640 with stable glutamine (ref L0498) and 100x streptomycin/penicillin solution (ref L0010) were obtained from Biowest. Foetal bovine serum (FBS, ref A3160502) and dialyzed FBS (ref 26400044) were obtained from Gibco. RPMI 1640 media without glucose, glutamine, and amino acids (ref R9010-01) were supplied by USBiological. U-^13^C-glucose, U-^13^C-glutamine and U-^13^C-glutamine were obtained from Cambridge Isotope Laboratories. FASN inhibitors GSK2194069 [6] and FADS2 inhibitor SC26196 [7] were purchased from Sigma-Aldrich; SCD inhibitor A93572 [8,9] was obtained from MedChemExpress. FA standards came from Sigma-Aldrich, Larodan and Cayman Chemicals. Antibodies anti-CD3 (ref BE0001-1) and anti-CD28 (ref BE0015-1) were provided by BioXCell. Recombinant IL-2 (ref 212-12) was obtained from Peprotech.

**Mice**

For all the experiments, 8-10-week-old female mice were used. 6-week-old wild-type C57BL/6 were purchased from Charles River Laboratories. Mice were left in a normal light cycle (08:00–20:00h) and had free access to water and a standard chow diet. Animals were housed in the Health Research Institute–Hospital La Fe Valencia facilities. Mouse studies followed the protocols approved by the Health Research Institute–Hospital La Fe Valencia Ethics and Animal Care and Use Committee (Protocol number 2020/VSC/PEA/0048).

Isolation, culture, and stimulation of mouse naïve CD8^+^ T-cells. To isolate naïve CD8^+^ T-cells, spleens were harvested. Single-cell suspensions were prepared by manual disruption and passage through a 70-μm cell strainer in PBS supplemented with 0.5% BSA and 2 mM EDTA. After RBC lysis, naïve CD8+ T-cells were purified by magnetic bead separation using commercially available kits following manufacturers’ instructions (naïve CD8a+ T-Cell Isolation Kit, mouse, Miltenyi Biotec Inc.) [10].

Cells were cultured in complete RPMI media (RPMI 1640 supplemented with 10% FBS, 100 U ml−1 penicillin, 100 μg ml−1 streptomycin, 55 μM 2-mercaptoethanol). Naïve T-cells were stimulated for 48 h with plate-bound anti-CD3 (10 μg ml−1) and anti-CD28 (5 μg ml−1) in complete RPMI media supplemented with recombinant IL-2 (100 U ml−1). All the experiments on ‘active’ T-cells were performed on day 4–5 postactivation [10].

**Cell lines and growth conditions**

The KRAS-mutant NSCLC cell line A549 was originally obtained from ATCC. The A549 cells were maintained in RPMI-1640 media supplemented with 10% FBS, 100 U ml−1 penicillin and 100 μg ml−1 streptomycin, and were routinely screened for mycoplasma contamination. Identity was confirmed by STR sequencing.

Cell metabolism studies. Isotopically-labelled media were prepared from glucose, glutamine and amino acids-free RPMI media, and were supplemented with 10% dialyzed FBS, 100 U/ml penicillin and 100 μg/ml streptomycin. For the culture of the CD8^+^ T-cells, media were also supplemented with recombinant IL-2 (100 U ml−1) and 55 μM 2-mercaptoethanol. U-^13^C-glucose and U-^13^C-glutamine were added at the normal concentration found in RPMI 1640 media. U-^13^C-acetate was added at 100 μM. U-^13^C-lactate was added at 11 mM [2,3,10–12].

The CD8^+^ T-cells were seeded at 0.8 x 10^6^ cells/mL and incubated for 72h with labelled media (and inhibitors). At 24h and 48h, cells were counted using the Countess II automated cell counter (Thermo Fischer Scientific) and density was adjusted to 0.8 x 10^6^ cells/mL with complete fresh labelled media (and inhibitors). At 72h, the final cell density was determined. Then, cells were transferred to 1.5 mL Eppendorf tubes and pelleted (500 g, 3 min). Media were removed. Cells were washed once with cold PBS 1x, resuspended in 500 μL of cold PBS 1x and stored at -80ºC [2,10].

For the NSCLC cell line A549, cells were seeded at 7 x 10^4^ cells/well in 6-well plates. After 24 h, media were replaced with labelled media (and inhibitors). Cells were incubated for 48-72 h until 80-90% confluence, the media was replaced with fresh media (and inhibitors) every 24h. At the end of the incubation, media were removed, cells were washed once with cold PBS 1x, scraped with 500 μL of cold PBS 1x, transferred to 1.5 mL Eppendorf tubes and stored at -80ºC [11].

**Saponification and extraction of total FAs**

To analyze the total FAs, 450 μL of cell suspension were transferred to a glass vial, and 1,000 μL of a 9:1 MeOH:KOH (3M in H2O) solution containing PC(16:0/16:0)D62 at 3 ppm were added. Saponification was performed for 1 h at 80 ºC in a water bath. After saponification, samples were cooled on ice and acidified by adding 100 μL of formic acid. FAs were extracted with 2 mL of heptane:isooctane (1:1) (2x), dried in a nitrogen flow, resuspended in 200 μL of mobile phase A containing myristic acid D27 at 1 ppm and transferred to a glass HPLC vial [13].

UPLC-HRMS analysis of FAs. FAs were analyzed in a quadrupole–orbitrap mass spectrometer (Q Exactive, Thermo-Fisher Scientific) coupled to reverse phase chromatography via electrospray ionization. Liquid chromatography separation was performed in a Cortecs C18 column (2.1 mm × 150 mm, 1.6 μm particle size; Waters). Solvent A was 2.5 mM ammonium acetate in 60:40 water:methanol. Solvent B was 2.5 mM ammonium acetate in 95:5 acetonitrile:isopropanol. The flow rate was 300 μL/min, the column temperature was 45ºC, the autosampler temperature was 5 ºC and the injection volume was 5 μL. The liquid chromatography gradient was: 0 min, 45% B; 0.5 min, 45% B; 19 min, 55% B; 23 min, 99% B; 34 min, 99% B. Between injections, the column was washed for 2 min with 50:50 acetonitrile:isopropanol before being equilibrated to the initial conditions. The mass spectrometer operated in the negative-ion mode to scan from *m/z* 100 to 450 at a resolving power of 140000. Data were acquired in the centroid mode.

Supplementary Figures


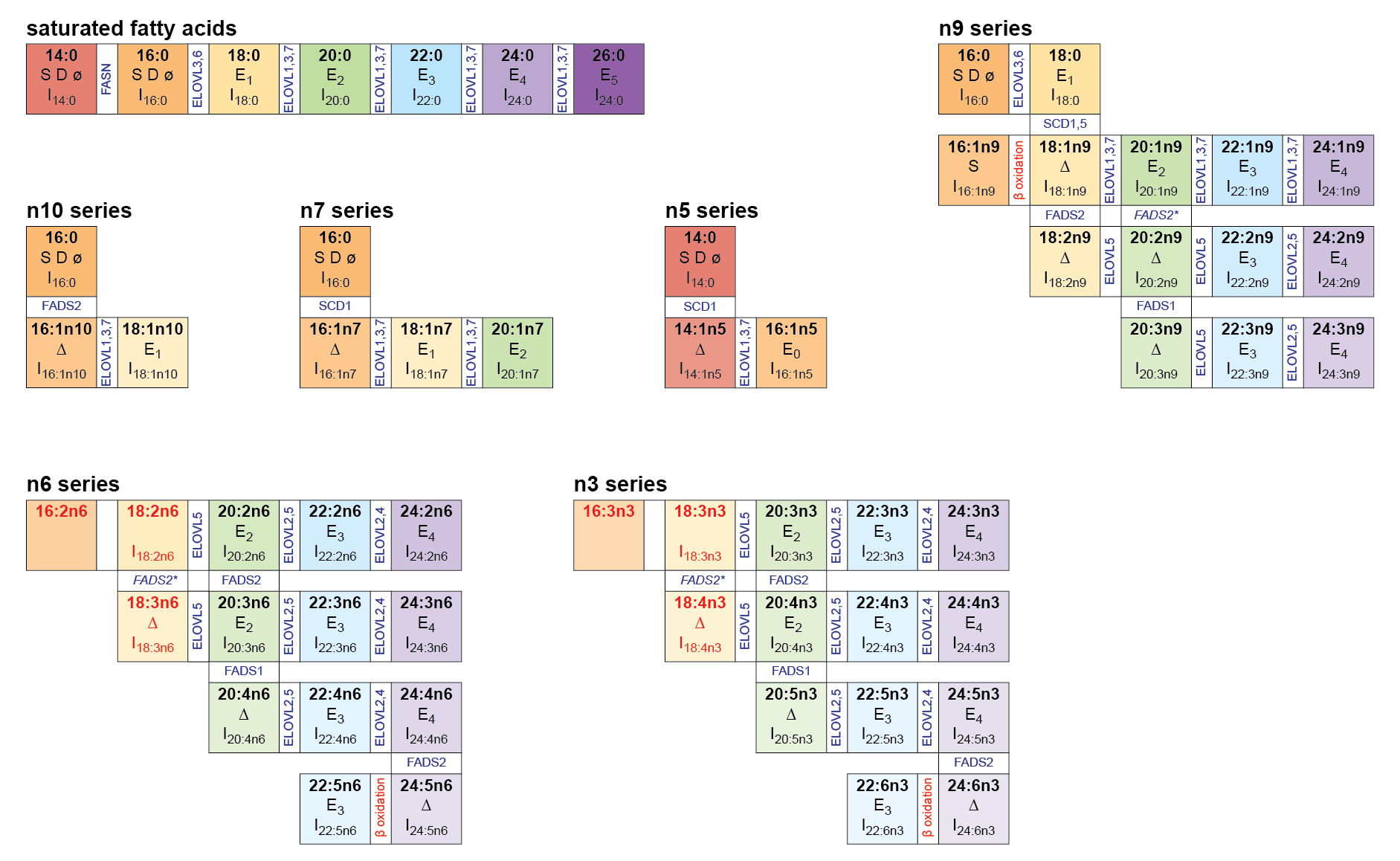


Supplementary Figure 1. FA metabolism network.

Summary of the FA interconversions covered by FAMetA and the parameters that can be estimated for each one. In red, the FA for which no parameter can be estimated because they are either solely imported or result from desaturation being performed on them. Horizontal transitions denote elongations and vertical transitions depict desaturations. The responsible enzymes are indicated in both cases. We assume DNL up to FA(16:0), although the calculation of the DNL parameters can be estimated for both FA(14:0) and FA(16:0). For the transformations of FA(18:2n6) into FA(20:3n6) and FA(18:3n3) to FA(20:4n3), the preferred route is desaturation, followed by elongation. The asterisk denotes a secondary route.


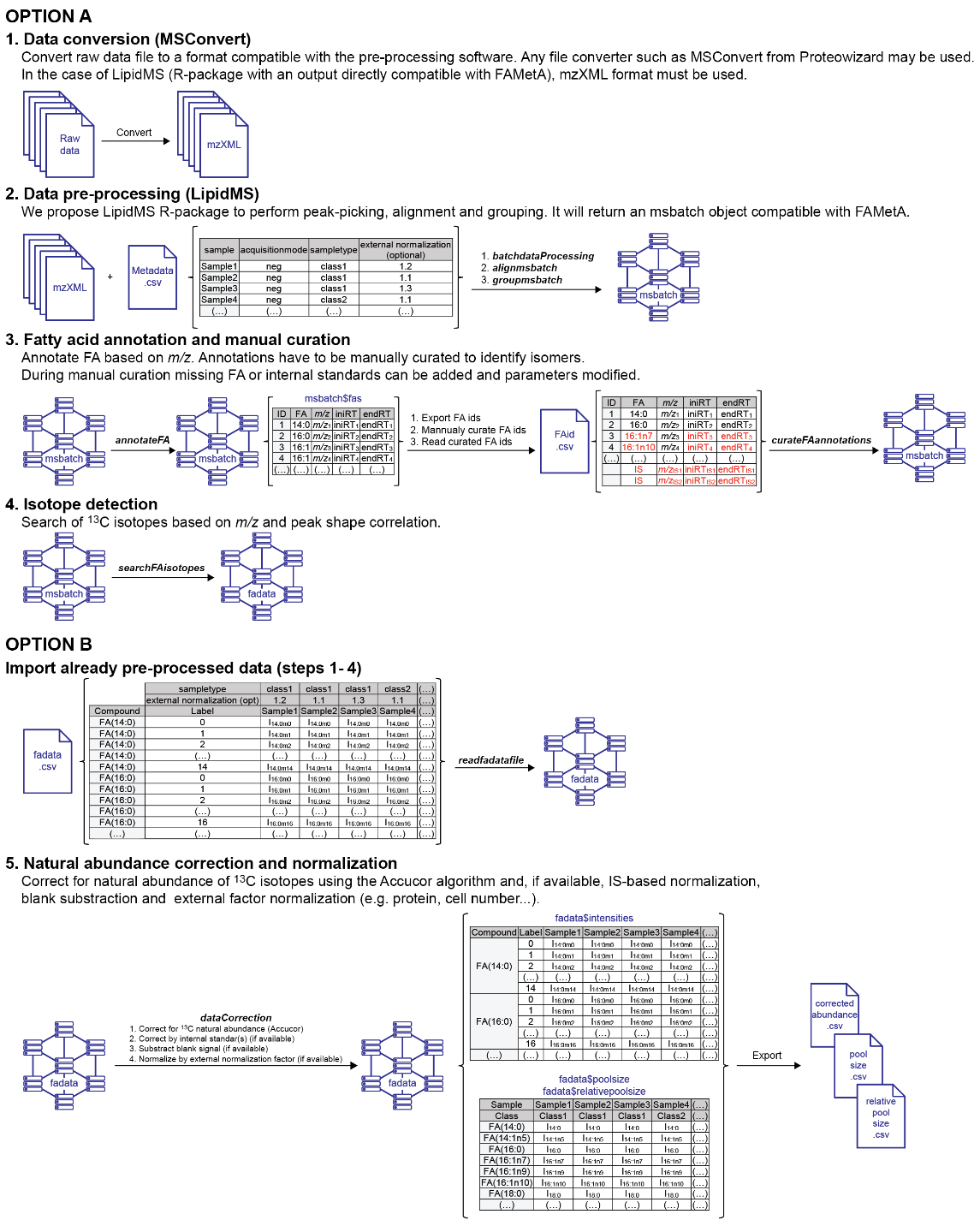


Supplementary Figure 2. Detailed workflow for data pre-processing.

Data pre-processing can be performed with a combination of our developed in-house R package LipidMS and FAMetA (Option A), or using any other suitable pre-processing tool (Option B). For data pre-processing with LipidMS, raw data files must firstly be converted into mzXML. LipidMS uses raw data files in the mzXML format and a csv metadata file as input to cover peak-peaking, alignment, grouping and peak filling. Output is a *msbatch* object that can be directly used by FAMetA to perform other pre-processing steps, including FA annotation and isotope detection. Output is a *fadata* object that can be used to conduct the final pre-processing step, which is natural abundance correction and normalisation. *Italics* depict the functions that can be executed in LipidMS or FAMetA.Type or paste caption here. Create a page break and paste in the Figure above the caption.


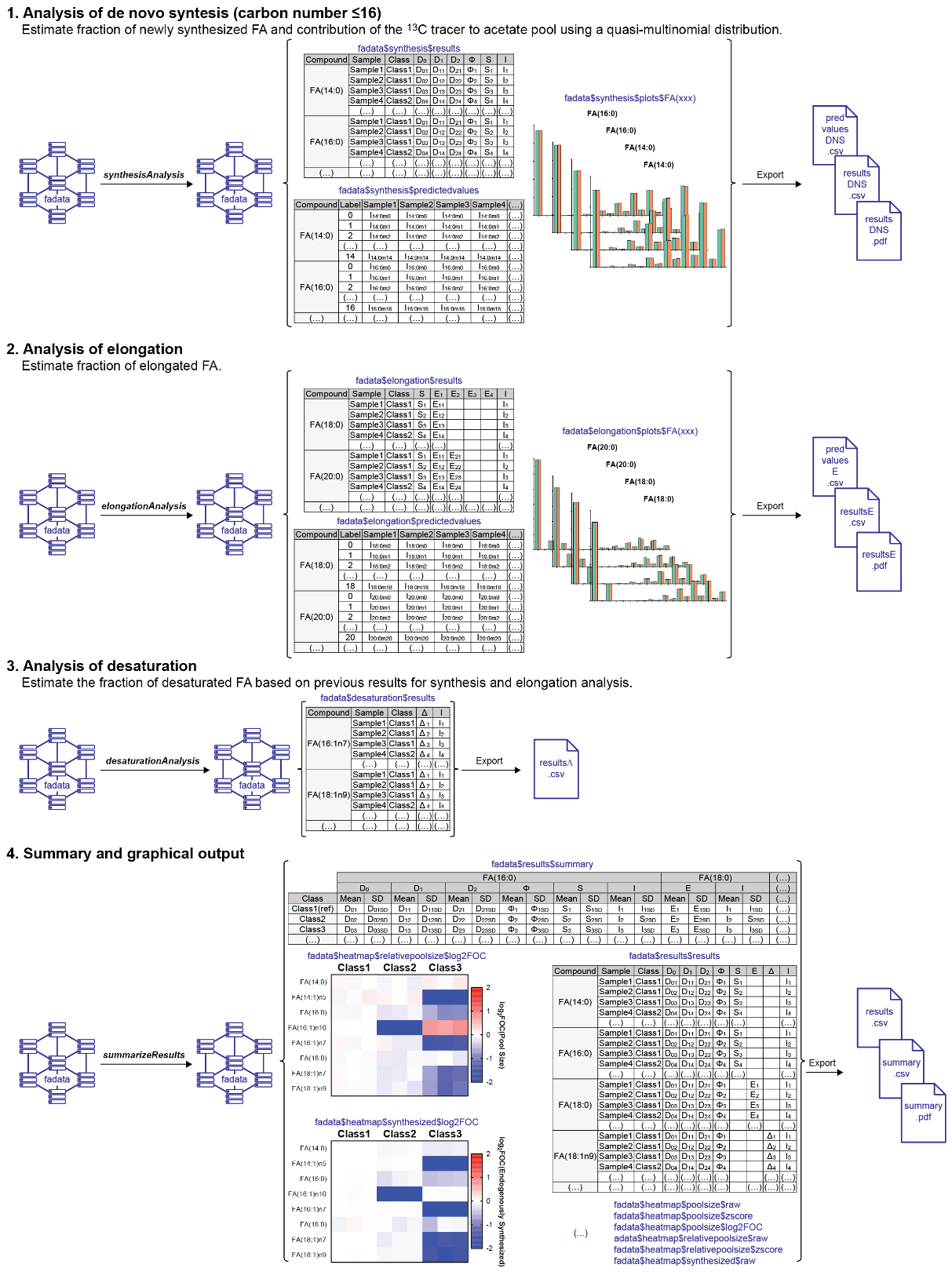


Supplementary Figure 3. Detailed FAMetA workflow and output.

Starting with the *fadata* object generated during data preprocessing (**Fig. S3**), FAMetA sequentially performs the analysis of DNS (*synthesisAnalysis* function), elongation (*elongationAnalysis* function) and desaturation (*desaturationAnalysis* function). The results for each step can be exported or a summary of all the calculated parameters and a group-based comparison can be obtained by executing the function *summarizeResults*.


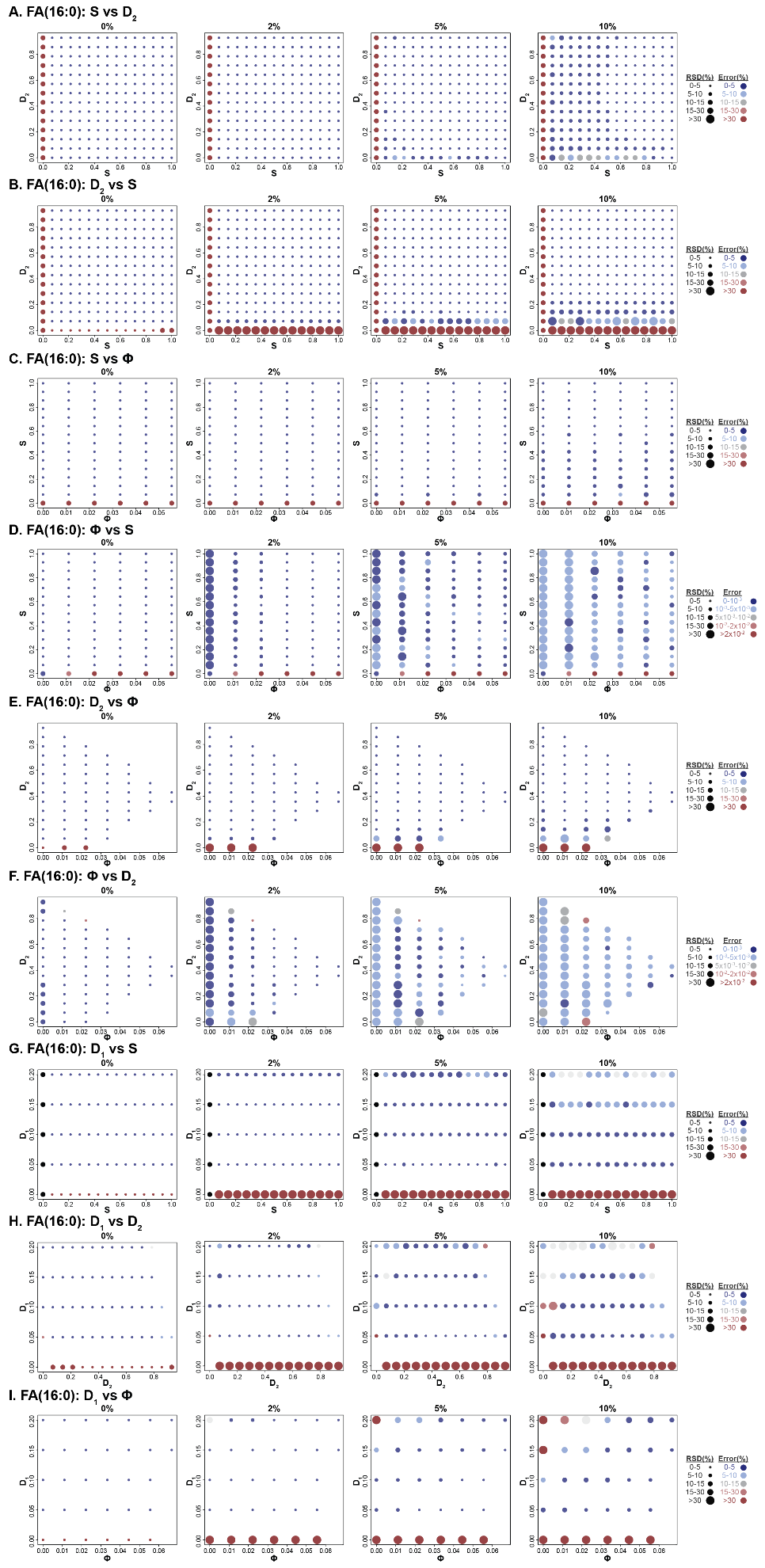


Supplementary Figure 4. *In silico* validation of the estimation of the *de novo* synthesis of FA(16:0).

To evaluate FAMetA’s ability to estimate the DNL analysis parameters, realistic values for *D_1_* (5 values from 0 to 0.2), *D_2_* (15 values from 0 to 1), *Φ* (10 points from 0 to 0.1) and *S* (15 values from 0 to 1) are combined to simulate 3,945 theoretical FA(16:0) distributions, to which the 0%, 2%, 5% and 10% noise levels are added to obtain 10 different noised distributions for each set of parameters. **A**, Evaluation of *S* as a function of *S* and *D_2_*. **B**, Evaluation of *D_2_* as a function of *S* and *D_2_*_._ **C**, Evaluation of *S* as a function of *S* and *Φ* (*D_2_*=0.5). **D**, Evaluation of *Φ* as a function of *S* and *Φ* (*D_2_*=0.5). **E**, Evaluation of *D_2_* as a function of *D_2_* and *Φ* (*S* =0.5). **F**, Evaluation of *Φ* as a function of *D_2_* and *Φ* (*S* =0.5). **G**, Evaluation of *D_1_* as a function of *D_1_* and *S*. **H**, Evaluation of *D_1_* as a function of *D_1_* and *D_2_*. **I**, Evaluation of *D_1_* as a function of *D_1_* and *Φ.*


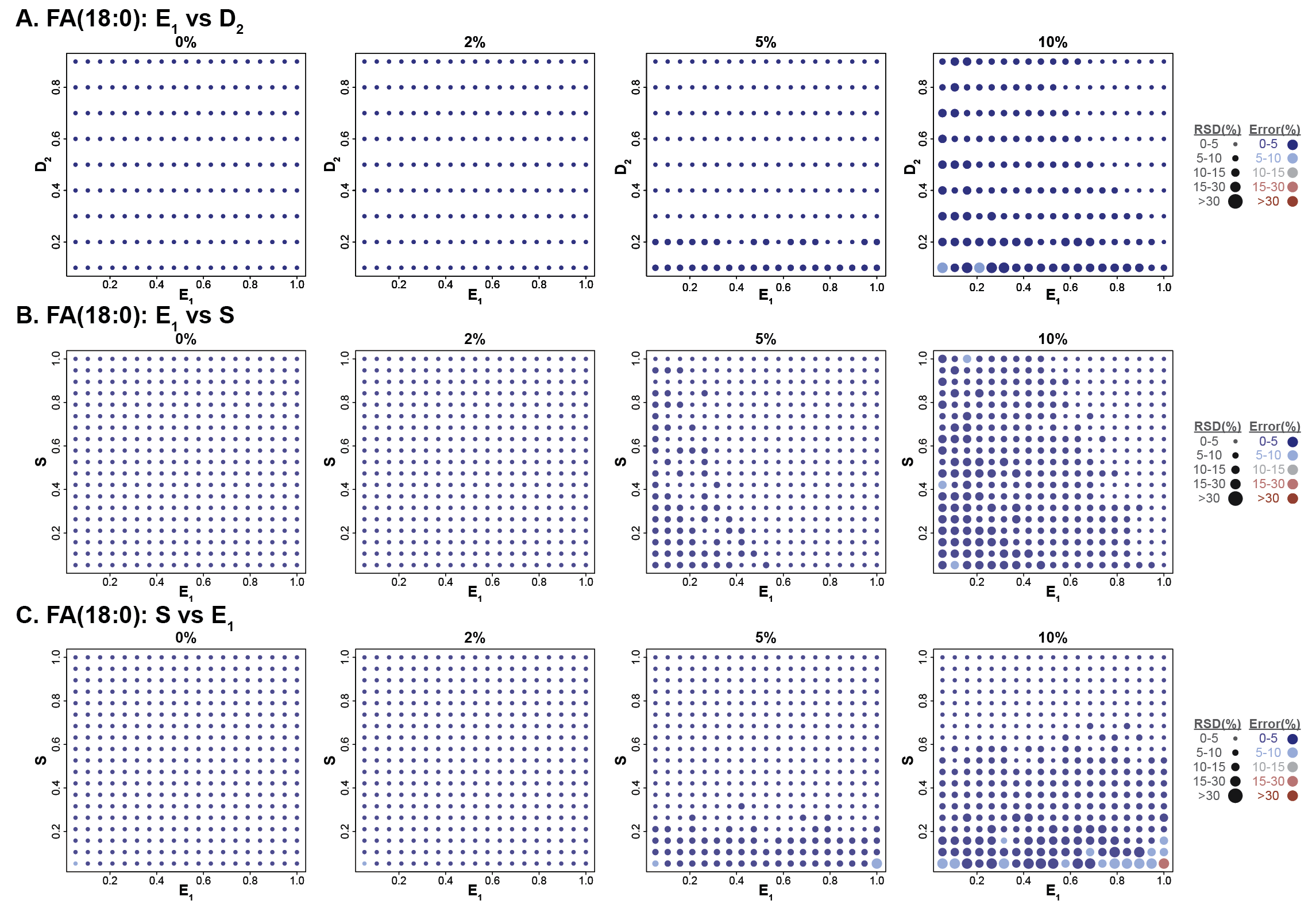


Supplementary Figure 5. *In silico* validation of the estimation of the *de novo synthesis* of FA(18:0).

To evaluate FAMetA’s ability to estimate parameters of elongation, the following values are set to simulate the mass-isotopologue data: *D_1_* and *Φ* are set at 0.05, and 0.01, respectively, *D_2_* varies from 0.1 to 0.9, and *E_1_* and *S* from 0.05 to 1. The 0%, 25, 5% and 10% noise levels are added to obtain 10 different noised distributions for each set of parameters. **A-B**, Evaluation of *E_1_* as a function of *E_1_* and *D_2_* (**A**), and *E_1_* and *S* (**B**). **C**, Evaluation of *S* as a function of *E_1_* and *S*.


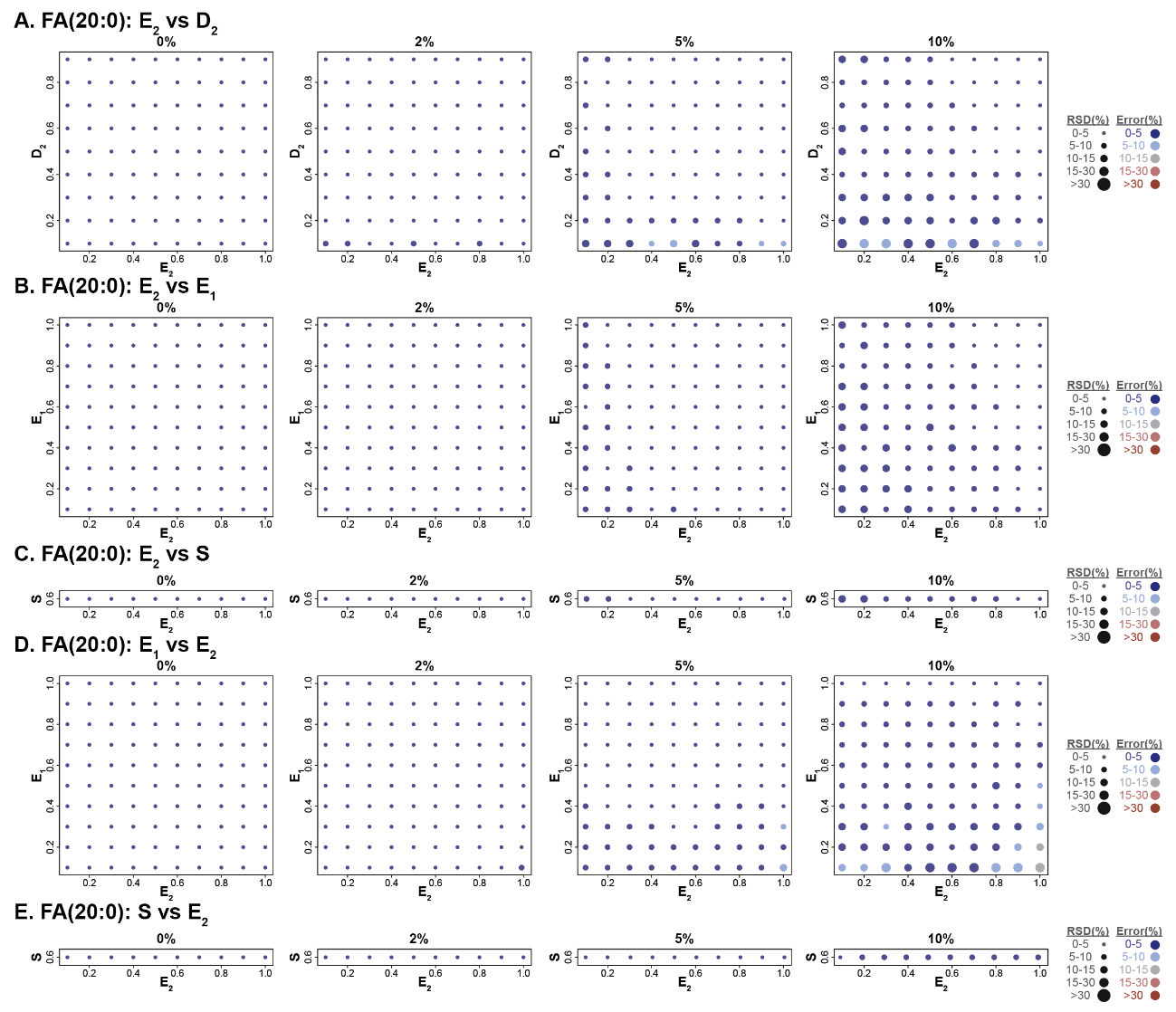


Supplementary Figure 6. *In silico* validation of the estimation of the *de novo synthesis* of FA(20:0).

To evaluate FAMetA’s ability to estimate parameters of elongation, the following values are set to simulate the mass-isotopologue data: *S*, *D_1_* and *Φ* are set at 0.6, 0.05 and 0.01, respectively, *D_2_* varies from 0.1 to 0.9, and *E_n_* from 0.1 to 1. The 0%, 2%, 5% and 10% noise levels are added to obtain 10 different noised distributions for each set of parameters. **A-C**, Evaluation of *E_2_* as a function of *E_2_* and *D_2_* (**A**), *E_2_* and *E_1_* (**B**), and *E_2_* and *S* (**C**). **D**, Evaluation of *E_1_* as a function of *E_2_* and *E_1_*. **E**, Evaluation of *S* as a function of *E_2_* and *S*.


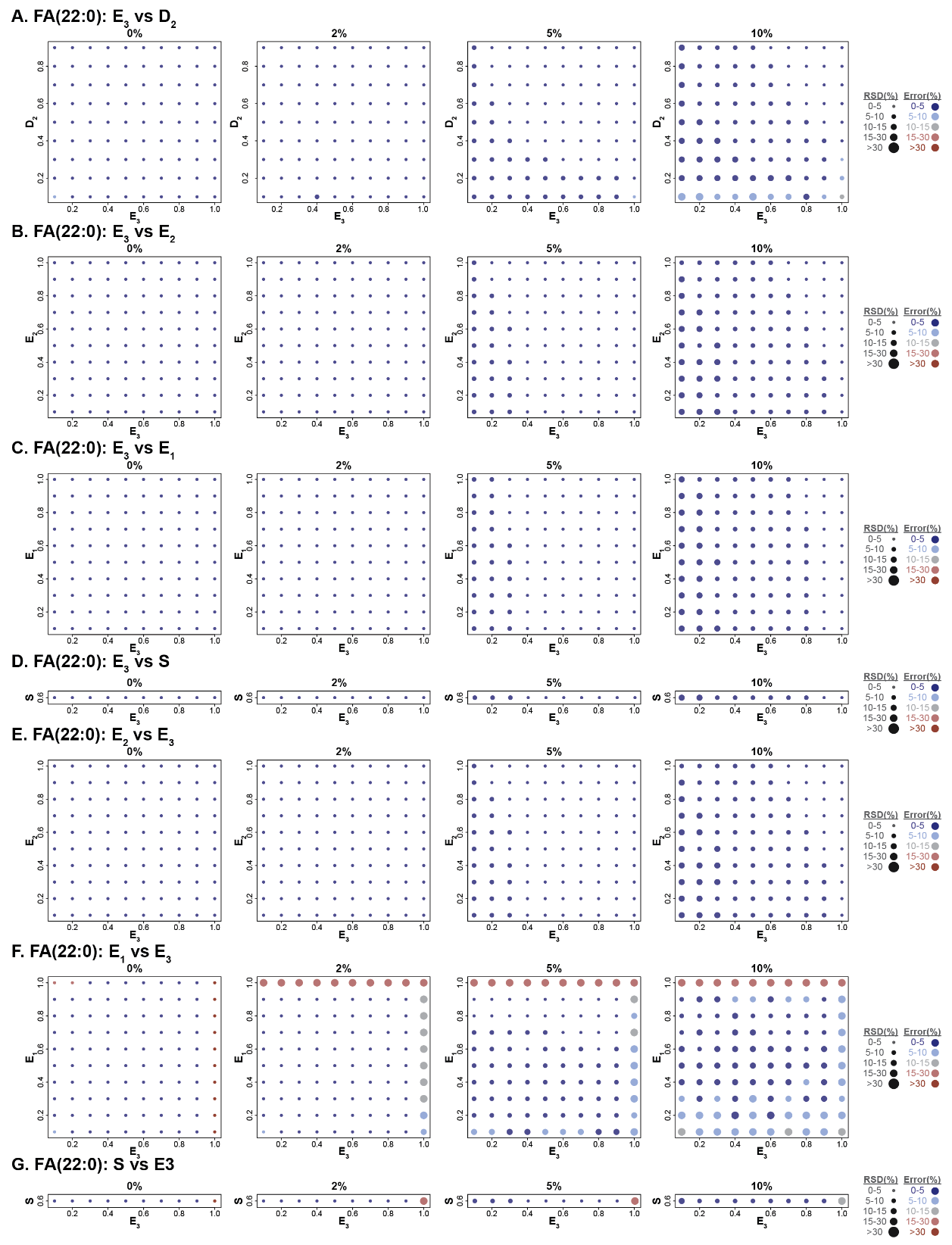


Supplementary Figure 7. *In silico* validation of the estimation of the *de novo synthesis* of FA(22:0).

To evaluate FAMetA’s ability to estimate parameters of elongation, the following values are set to simulate the mass-isotopologue data: *S*, *D_1_* and *Φ* are set at 0.6, 0.05 and 0.01, respectively, *D_2_* varies from 0.1 to 0.9, and *E_n_* from 0.1 to 1. The 0%, 2%, 5% and 10% noise levels are added to obtain 10 different noised distributions for each set of parameters. **A-D**, Evaluation of *E_3_* as a function of *E_3_* and *D_2_* (**A**), *E_3_* and *E_2_* (**B**), *E_3_* and *E_1_* (**C**), and *E_3_* and *S* (**D**). **E**, Evaluation of *E_2_* as a function of *E_3_* and *E_2_*. **F**, Evaluation of *E_1_* as a function of *E_3_* and *E_1_*. **G**, Evaluation of *S* as a function of *E_3_* and *S*.


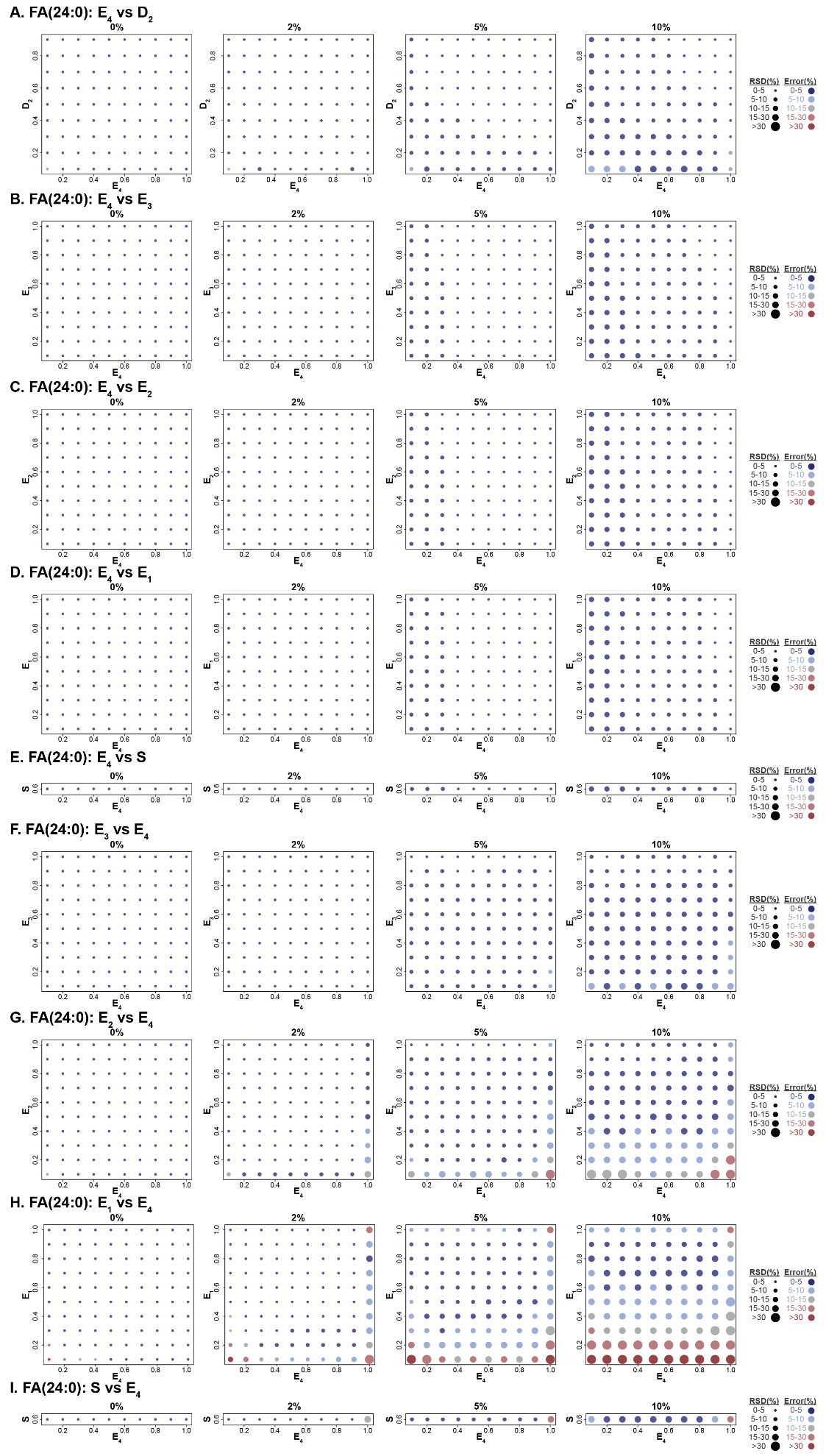


Supplementary Figure 8. *In silico* validation of the estimation of the *de novo synthesis* of FA(24:0).

To evaluate FAMetA’s ability to estimate parameters of elongation, the following values are set to simulate the mass-isotopologue data: *S*, *D_1_* and *Φ* are set at 0.6, 0.05 and 0.01, respectively, *D_2_* varies from 0.1 to 0.9, and *E_n_* from 0.1 to 1. The 0%, 2%, 5% and 10% noise levels are added to obtain 10 different noised distributions for each set of parameters. **A-E**, Evaluation of *E_4_* as a function of *E_4_* and *D_2_* (**A**), *E_4_* and *E_3_* (**B**), *E_4_* and *E_2_* (**C**), *E_4_* and *E_1_* (**D**), and *E_3_* and *S* (**E**). **F**, Evaluation of *E_3_* as a function of *E_4_* and *E_3_*. **G**, Evaluation of *E_2_* as a function of *E_4_* and *E_2_*. **H**, Evaluation of *E_1_* as a function of *E_4_* and *E_1_.* **I**, Evaluation of *S* as a function of *E_4_* and *S*.


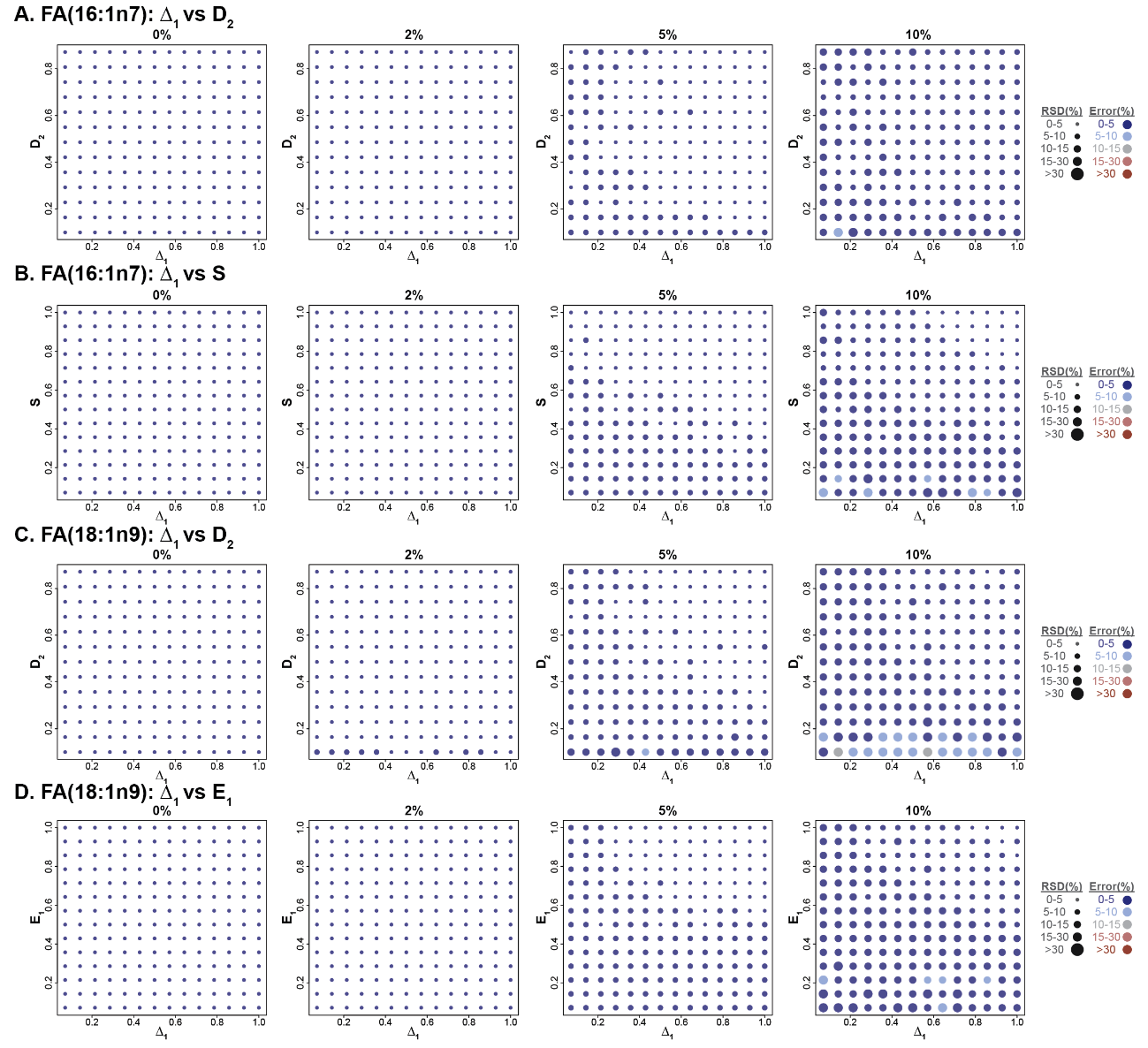


Supplementary Figure 9. *In silico* validation of the estimation of the *de novo synthesis* of FA(16:1n7) and FA(18:1n9).

To evaluate FAMetA’s ability to estimate parameters of desaturation, the following values are set to simulate the mass-isotopologue data: *D_1_* and *Φ* are set at 0.05 and 0.01, respectively, *D_2_* varies from 0.1 to 0.9, Δ_1_ varies from 0 to 1; for FA(16:1n7), S varies from 0.1 to 1; for FA(18:1n9) S is set at 0.6 and *E_1_* varies from 0.1 to 1. The 0%, 2%, 5% and 10% noise levels are added to obtain 10 different noised distributions for each set of parameters. **A-B**, Evaluation of Δ_1_ for FA(16:1n7) as a function of *D_2_* and Δ_1_ (**A**) or *S* and Δ_1_ (**C**). **C-D**, Evaluation of Δ_1_ for FA(18:1n9) as a function of *D_2_* and Δ_1_ (**C**) or *E_1_* and Δ_1_ (**D**).
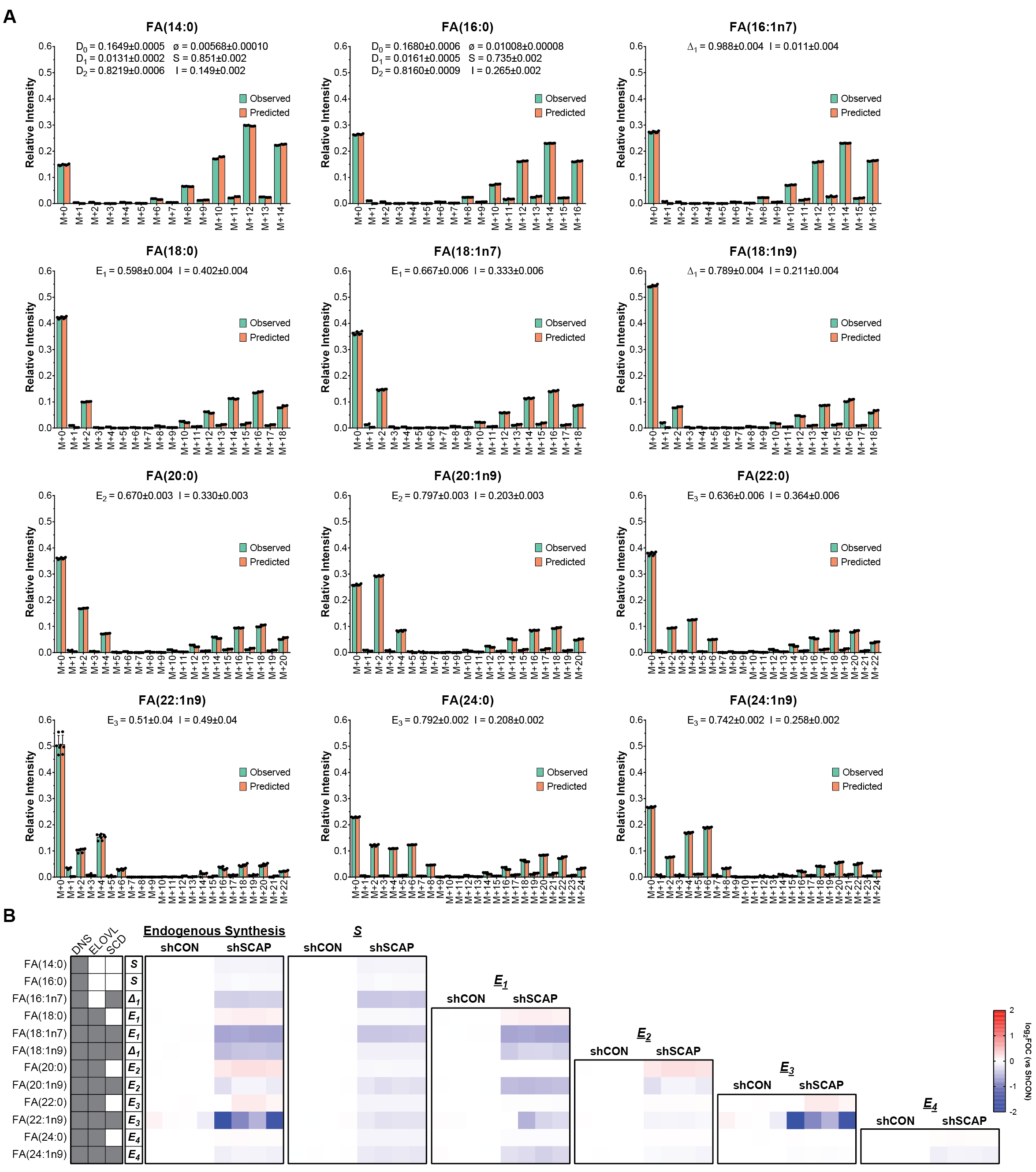


Supplementary Figure 10. Analysis of the influence of the down-regulation of SCAP on the FA metabolism in the H1299 cells; data obtained from ref [4]

**A**, FAMetA was used to fit all the reported experimental mass-isotopologue distributions for the control condition (shCON). **B**, Heatmap showing the log_2_ fold of change (vs. shCON) for each reported FA in the following parameters: endogenously synthesized fraction, calculated *S*, *E_1_*, *E_2_*, *E_3_* and *E_4_*. For each FA, the parameter reported for endogenous synthesis is indicated. The shadowed cells indicate the activities (DNS, elongation (ELOVL), or SCD1-mediated desaturation (SCD)) involved in the synthesis of a particular FA. n=4.


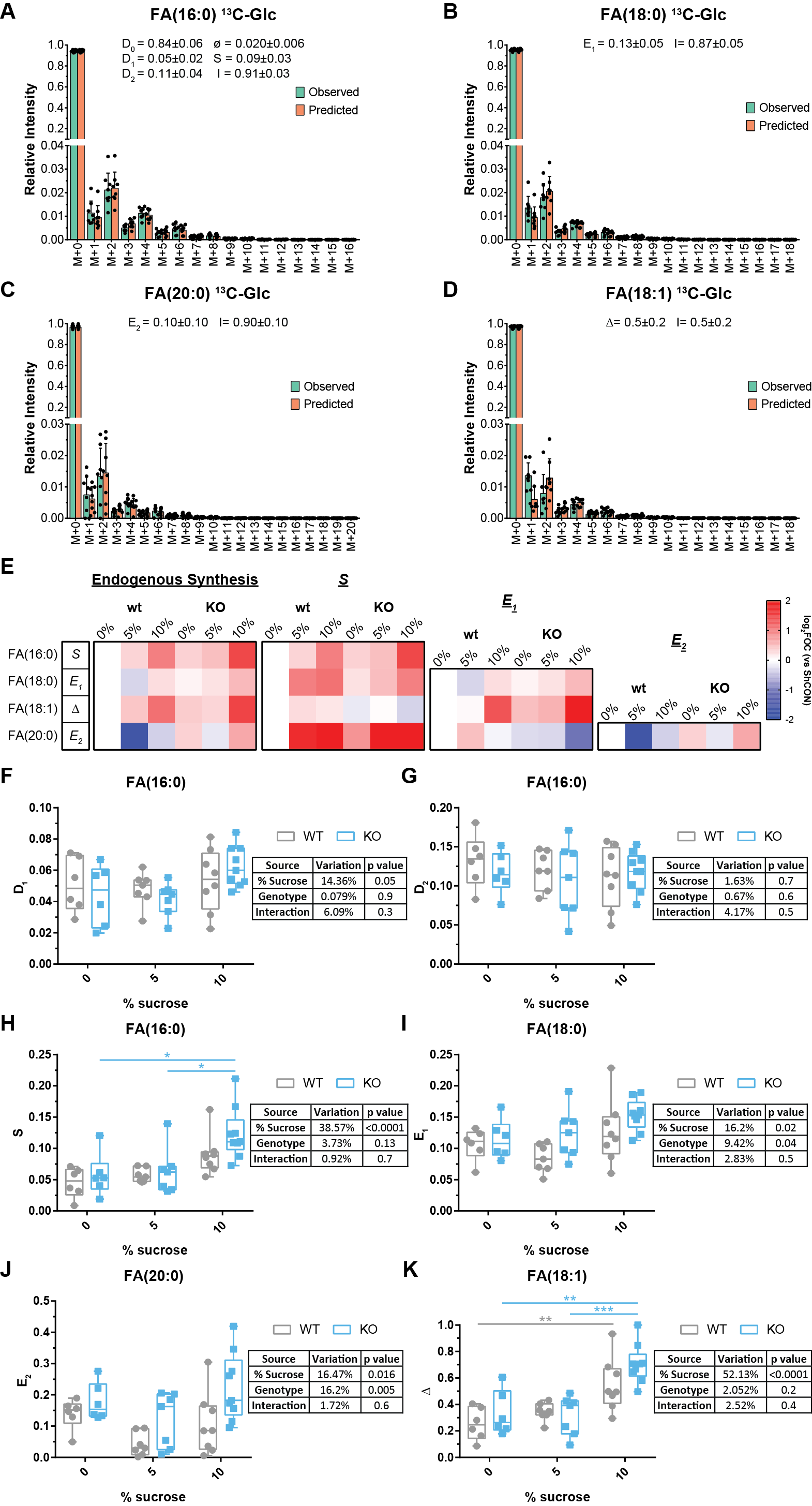


Supplementary Figure 11. Effect of KHK-C expression and dietary sucrose on FA synthesis *in vivo*, data obtained from ref. [5]

**A-D,** FAMetA was used to fit all the reported experimental mass-isotopologue distributions for the WT 10% sucrose group. **E**, Heatmap showing the log_2_ fold of change (vs. WT 0% sucrose) for each reported FA in the following parameters: endogenously synthesized fraction, calculated *S*, *E_1_* and *E_2_*. For each FA, the parameter reported for the endogenous synthesis is indicated. The shadowed cells indicate the activities [DNS, elongation (ELOVL) or SCD1-mediated desaturation (SCD)] involved in the synthesis of a particular FA. **F-K**, The calculated FA synthesis parameters obtained with FAMetA. The tables summarize the result of the two-way ANOVA performed for each calculated parameter. Paired differences are calculated by a post hoc Tukey test. The p-values obtained for the reported significant differences: *S* parameter for FA(16:0), KO 10% vs. KO 0%, p-value =0.014, KO 10% vs. KO 5%, p-value =0.03; *Δ* parameter for FA(18:1), WT 10% vs. WT 0%, p-value =0.006, KO 10% vs. KO 0%, p-value =0.0012, KO 10% vs. KO 5%, p-value =0.0004 (n=6,6,7,7,8,8).

**
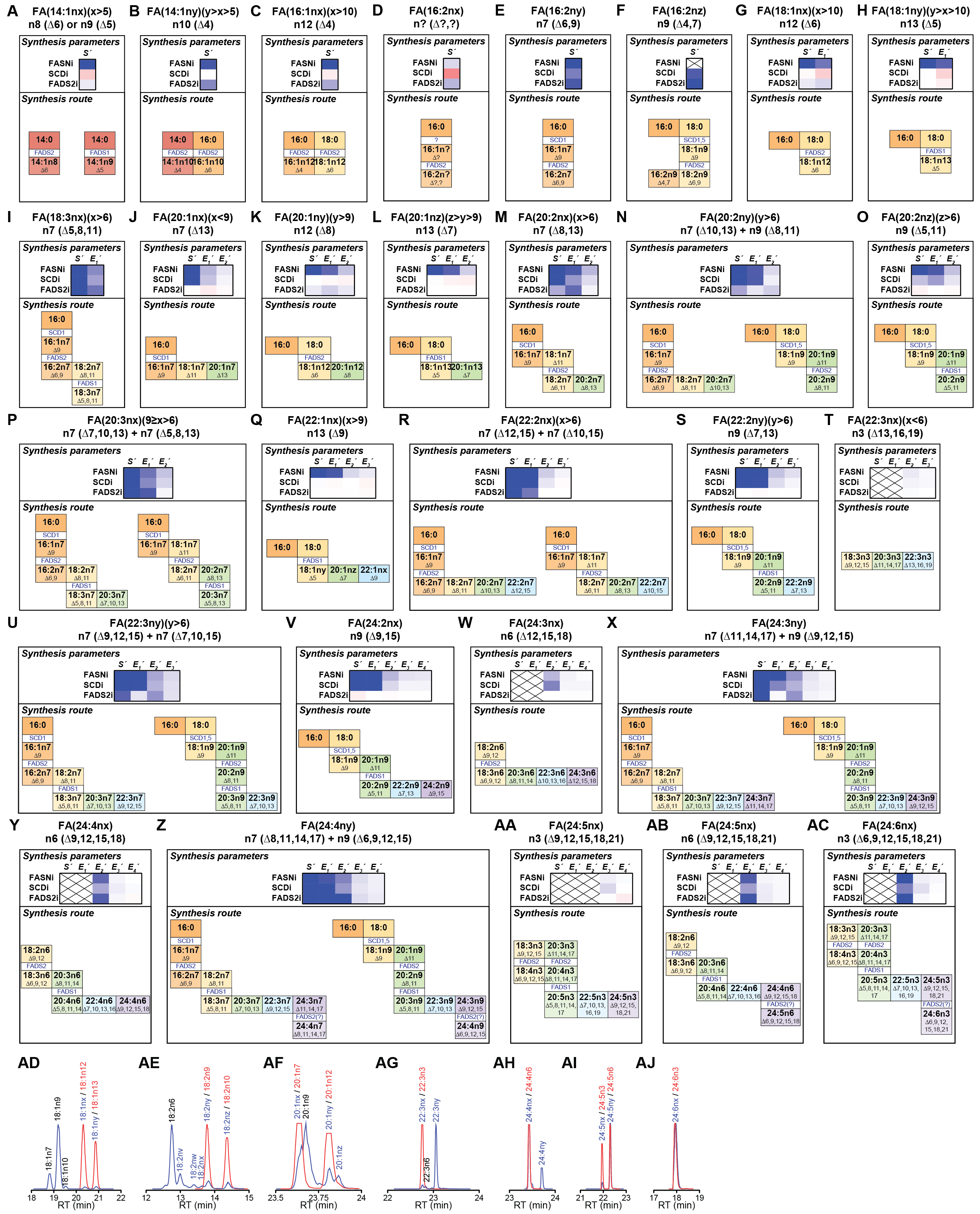
**

Supplementary Figure 12. Proposed synthesis route for the unknown FAs detected in A549 cells

**A-AC**, Mean value of the log_2_ fold-of-change (vs. untreated) in the synthesis parameters and the proposed synthesis route for FA(14:1nx) (**A**), FA(14:1ny) (**B**), FA(16:1nx) (**C**), FA(16:2nx) (**D**), FA(16:2ny) (**E**), FA(16:2nz) (**F**), FA(18:1nx) (**G**), FA(18:1ny), (**H**), FA(18:3nx) (**I**), FA(20:1nx) (**J**), FA(20:1ny) (**K**), FA(20:1nz) (**L**), FA(20:2nx) (**M**), FA(20:2ny) (**N**), FA(20:2nz) (**O**), FA(20:3nx) (**P**), FA(22:1nx) (**Q**), FA(22:2nx) (**R**), FA(22:2ny) (**S**), FA(22:3nx) (**T**), FA(22:3ny) (**U**), FA(24:2nx) (**V**), FA(24:3nx) (**W**), FA(24:3ny) (**X**), FA(24:4nx) (**Y**), FA(24:4ny) (**Z**), FA(24:5nx) (**AA**), FA(24:5ny) (**AB**) and FA(24:6nx) (**AC**). On heatmaps, crosses indicate missing or NA values. In the synthesis route description, horizontal transitions denote elongations (enzymes not indicated). Vertical transitions depict desaturations (enzymes indicated). **AD-AJ**, Confirmation of the identity of 11 unknown FAs in the A549 cells with chemical standards. Chromatographic separation of FAs 18:1 (**AD**), 18:2 (**AE**), 20:1 (**AF**), 22:3 (**AG**), 24:4 (**AH**), 24:5 (**A**I) and 24:6 (**AJ**). In blue, the saponified FAs from the A549 cells in culture. In red, chemical standards. Text in black, the FAs that initially matched the chemical standards used to develop the method; in blue, a notation of the unknown FAs detected in the A549 cells; in red, the chemical standards used to confirm the identity of the selected unknown FAs.

Supplementary Tables

Supplementary Table 1. Comparison of the features implemented within the main available tools for the analysis of FA metabolism.

|  | **ISA** | **ConvISA** | **Kamphorst** | **FASA** | **FAMetA** |
| --- | --- | --- | --- | --- | --- |
| De novo lipogenesis | 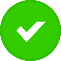 | 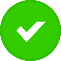 | 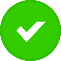 | 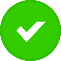 | 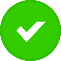 |
| Contribution of labeled nutrient to lipogenic AcetylCoA pool | 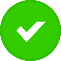 | 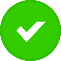 | 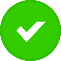 | 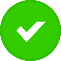 | 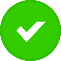 |
| Elongation | 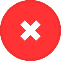 | 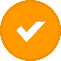 | 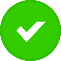 | 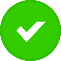 | 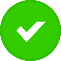 |
| Desaturation | 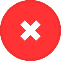 | 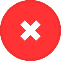 | 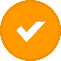 | 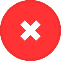 | 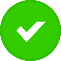 |
| Data pre-processing | 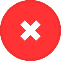 | 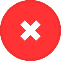 | 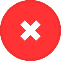 | 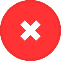 | 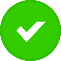 |
| Graphical output | 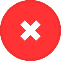 | 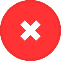 | 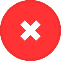 | 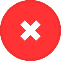 | 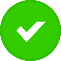 |
| Implementation | Matlab | Matlab  script | Matlab  script | Matlab  toolbox | R-package  Web-based app |
| Comments | The actual algorithm is not released as script or equivalent, but has to be implemented by users or used within a metabolic flux tool | Elongation calculated only for FA(18:0) | Steady state must be achieved as M+0 = import.  Desaturation based on total labeling and exemplified only for FA(18:1n9). | Elongation described as de novo lipogenesis up to the total number of carbons plus multiple import-elongation terms. |  |

Supplementary References

1. Metallo CM, Gameiro PA, Bell EL, et al. Reductive glutamine metabolism by IDH1 mediates lipogenesis under hypoxia. Nature 2012; 481:380–384

2. Garcia-Canaveras JC, Heo D, Trefely S, et al. CAR T-Cells Depend on the Coupling of NADH Oxidation with ATP Production. Cells 2021; 10:2334

3. Qiu J, Villa M, Sanin DE, et al. Acetate Promotes T Cell Effector Function during Glucose Restriction. Cell Rep. 2019; 27:2063-2074.e5

4. Argus JP, Wilks MQ, Zhou QD, et al. Development and Application of FASA, a Model for Quantifying Fatty Acid Metabolism Using Stable Isotope Labeling. Cell Rep. 2018; 25:2919-2934.e8

5. Jang C, Wada S, Yang S, et al. The small intestine shields the liver from fructose-induced steatosis. Nat. Metab. 2020; 2:586–593

6. Hardwicke MA, Rendina AR, Williams SP, et al. A human fatty acid synthase inhibitor binds β-ketoacyl reductase in the keto-substrate site. Nat. Chem. Biol. 2014; 10:774–779

7. Obukowicz MG, Raz A, Pyla PD, et al. Identification and characterization of a novel Δ6/Δ5 fatty acid desaturase inhibitor as a potential anti-inflammatory agent. Biochem. Pharmacol. 1998; 55:1045–1058

8. Xin Z, Zhao H, Serby MD, et al. Discovery of piperidine-aryl urea-based stearoyl-CoA desaturase 1 inhibitors. Bioorganic Med. Chem. Lett. 2008; 18:4298–4302

9. Von Roemeling CA, Marlow LA, Wei JJ, et al. Stearoyl-CoA desaturase 1 is a novel molecular therapeutic target for clear cell renal cell carcinoma. Clin. Cancer Res. 2013; 19:2368–2380

10. Ghergurovich JM, García-Cañaveras JC, Wang J, et al. A small molecule G6PD inhibitor reveals immune dependence on pentose phosphate pathway. Nat. Chem. Biol. 2020; 16:731–739

11. García-Cañaveras JC, Lancho O, Ducker GS, et al. SHMT inhibition is effective and synergizes with methotrexate in T-cell acute lymphoblastic leukemia. Leukemia 2021; 35:377–388

12. Kamphorst JJ, Chung MK, Fan J, et al. Quantitative analysis of acetyl-CoA production in hypoxic cancer cells reveals substantial contribution from acetate. Cancer Metab. 2014; 2:1–8

13. Kamphorst JJ, Fan J, Lu W, et al. Liquid chromatography-high resolution mass spectrometry analysis of fatty acid metabolism. Anal. Chem. 2011; 83:9114–9122
